# Bruton’s Tyrosine Kinase Inhibitors Impair FcγRIIA-Driven Platelet Responses to Bacteria in Chronic Lymphocytic Leukemia

**DOI:** 10.3389/fimmu.2021.766272

**Published:** 2021-11-29

**Authors:** Leigh Naylor-Adamson, Anisha R. Chacko, Zoe Booth, Stefano Caserta, Jenna Jarvis, Sujoy Khan, Simon P. Hart, Francisco Rivero, David J. Allsup, Mònica Arman

**Affiliations:** ^1^ Centre for Atherothrombosis and Metabolic Disease, Hull York Medical School, Faculty of Health Sciences, University of Hull, Hull, United Kingdom; ^2^ Department of Biomedical Sciences, Faculty of Health Sciences, University of Hull, Hull, United Kingdom; ^3^ Department of Immunology & Allergy, Queens Centre, Castle Hill Hospital, Hull University Teaching Hospitals NHS Trust, Cottingham, United Kingdom; ^4^ Respiratory Research Group, Hull York Medical School, Faculty of Health Sciences, University of Hull, Hull, United Kingdom; ^5^ Department of Haematology, Queens Centre for Oncology and Haematology, Castle Hill Hospital, Hull University Teaching Hospitals NHS Trust, Cottingham, United Kingdom

**Keywords:** platelet, FcγRIIA, *Staphylococcus aureus*, *Escherichia coli*, Bruton’s tyrosine kinase inhibitor, ibrutinib, chronic lymphocytic leukemia (CLL)

## Abstract

Bacterial infections are a major cause of morbidity and mortality in chronic lymphocytic leukemia (CLL), and infection risk increases in patients treated with the Bruton’s tyrosine kinase (Btk) inhibitor, ibrutinib. Btk and related kinases (like Tec) are expressed in non-leukemic hematopoietic cells and can be targeted by ibrutinib. In platelets, ibrutinib therapy is associated with bleeding complications mostly due to off-target effects. But the ability of platelets to respond to bacteria in CLL, and the potential impact of ibrutinib on platelet innate immune functions remain unknown. FcγRIIA is a tyrosine kinase-dependent receptor critical for platelet activation in response to IgG-coated pathogens. Crosslinking of this receptor with monoclonal antibodies causes downstream activation of Btk and Tec in platelets, however, this has not been investigated in response to bacteria. We asked whether ibrutinib impacts on FcγRIIA-mediated activation of platelets derived from CLL patients and healthy donors after exposure to *Staphylococcus aureus* Newman and *Escherichia coli* RS218. Platelet aggregation, α-granule secretion and integrin αIIbβ3-dependent scavenging of bacteria were detected in CLL platelets but impaired in platelets from ibrutinib-treated patients and in healthy donor-derived platelets exposed to ibrutinib *in vitro*. While levels of surface FcγRIIA remained unaffected, CLL platelets had reduced expression of integrin αIIbβ3 and GPVI compared to controls regardless of therapy. In respect of intracellular signaling, bacteria induced Btk and Tec phosphorylation in both CLL and control platelets that was inhibited by ibrutinib. To address if Btk is essential for platelet activation in response to bacteria, platelets derived from X-linked agammaglobulinemia patients (lacking functional Btk) were exposed to *S. aureus* Newman and *E. coli* RS218, and FcγRIIA-dependent aggregation was observed. Our data suggest that ibrutinib impairment of FcγRIIA-mediated platelet activation by bacteria results from a combination of Btk and Tec inhibition, although off-target effects on additional kinases cannot be discarded. This is potentially relevant to control infection-risk in CLL patients and, thus, future studies should carefully evaluate the effects of CLL therapies, including Btk inhibitors with higher specificity for Btk, on platelet-mediated immune functions.

## Introduction

Chronic lymphocytic leukemia (CLL) is the most common form of leukemia in the Western World, with approximately 3750 new cases diagnosed annually in the United Kingdom (1). Infectious complications are a major cause of morbidity and mortality in CLL patients (2, 3). The increased risk of infection is due to multiple factors including inherent disease-related immune dysfunction due to secondary hypogammaglobulinemia and T-lymphocyte dysfunction; patient-related factors like age, frailty and co-morbidities; and therapy-related immunosuppression (4).

Bruton’s tyrosine kinase (Btk) is a member of the Tec family of cytoplasmic tyrosine kinases, with a primary role in CLL pathogenesis through signaling downstream of the B cell receptor (BCR) (5). The first-generation inhibitor of Btk (iBtk), ibrutinib, is an effective therapeutic strategy for CLL but has significant toxicity, particularly related to infections and hemorrhagic complications (5–8), due to inhibition of Btk expressed in other (non-B lymphocyte) hematopoietic cells and off-target effects on other kinases (9–11). Bleeding complications are linked to ibrutinib inhibition of platelet responses to multiple agonists that specifically signal through tyrosine kinase-linked receptors, including GPVI, GPIb-IX-V, CLEC-2, and integrins αIIbβ3 and α2β1 (12–16). Healthy individuals and CLL patients can be classified in two groups characterized by low or high platelet sensitivity to ibrutinib based on collagen-induced platelet aggregation *in vitro*, which is related to drug efflux pumps (17). However, a recent study suggests that ibrutinib-dependent bleeding in CLL patients also involves underlying disease-related changes in platelets including decreased platelet count and impaired platelet response to ADP (18).

Platelets play important immune functions during infection like scavenging and containment of bacteria, secretion of antimicrobial substances and interaction with leukocytes (19–24). In CLL, alterations in the innate immune system have been reported, including impaired function of neutrophils, natural killer cells and monocyte/macrophages (4, 25, 26), however, platelet immune functions remain uncharacterized. Moreover, the potential effect of iBtk therapy on platelet responses to infection has not been addressed. *Streptococcus pneumoniae* and *Haemophilus influenzae* are predominant pathogens in CLL, however, ibrutinib treatment has also been associated with serious infections by *Staphylococcus aureus* and *Escherichia coli* (3, 6, 27, 28). Importantly, most of these bacterial species are known to cause platelet activation (29–32). When platelets encounter bacteria, contact among them usually involves multiple bacterial strain-specific interactions with different platelet receptors [e.g., FcγRIIA (also known as CD32a), αIIbβ3, GPIb, complement receptor gC1q-R, and Toll-like receptor 2] (33, 34). Although each one of these molecular interactions can contribute to the adhesion and/or platelet activation steps, FcγRIIA has a central role in triggering final platelet activation in response to a wide range of bacteria (29–31).

FcγRIIA recognizes IgG-opsonized pathogens and signals *via* its cytoplasmic immunoreceptor tyrosine-based activation motif domain (30). Ligation of FcγRIIA by antibody crosslinking causes phosphorylation of Btk and Tec in healthy donor platelets (35) and leads to platelet activation that can be inhibited by iBtks (36). However, activation of FcγRIIA by bacteria is different from crosslinking the receptor with antibodies (29–31). Distinct features of the former include the presence of a lag phase between stimulation and onset of aggregation, and the fact that FcγRIIA phosphorylation and platelet secretion depend on integrin αIIbβ3 engagement (29, 31). Therefore, it is necessary to study the effect of iBtks on platelet FcγRIIA activation following exposure to pathophysiological stimuli including bacteria.

In this study, we analyze if platelets from CLL patients can respond to bacteria in an FcγRIIA-dependent manner and investigate the hypothesis that ibrutinib impairs such responses potentially contributing to the increased risk of infection reported in CLL patients treated with this drug.

## Material and Methods

### Reagents

See **Supplementary Information** for details.

### Bacterial Culture and Preparation

*S. aureus* Newman (a gift from Prof Steve Kerrigan, RCSI, Ireland) and *E. coli* RS218 (a gift from Prof Ian Henderson, University of Queensland, Australia) were cultured and prepared as described (29, 31) (**Supplementary Information**).

### Human Samples and Ethical Considerations

This study was performed in accordance with relevant ethics committees: Hull York Medical School (reference number 1501) and UK National Health Service Research Ethics (08/H1304/35). Informed consent was obtained from all participants.

Peripheral blood from CLL and X-linked agammaglobulinemia (XLA) patients was taken at the Departments of Haematology and Immunology & Allergy, respectively, at Castle Hill Hospital (Cottingham, UK). Blood was drawn using sodium citrate or acid-citrate-dextrose (ACD) vacutainers (see below), and shipped to the University of Hull within 4 hours of venepuncture for immediate testing. Ibrutinib-treated CLL patients were taking a daily dose of 420 mg, except for two patients who were taking 140 mg. Blood from healthy donors was collected at the University of Hull in syringes containing sodium citrate or ACD from volunteers over 18 years of age not treated with concurrent anti-platelet agents.

Platelet-rich plasma (PRP) and platelet-poor plasma (PPP) were obtained from sodium citrated blood, while washed platelets (WP) were prepared from blood in ACD, as detailed in **Supplementary Information**.

### Platelet Aggregation

Platelet aggregation in PRP or WP was done under stirring conditions (1000 rpm) at 37°C using light transmission aggregometry with a CHRONO-LOG 490 aggregometer (CHRONO-LOG, Havertown, PA, USA). WP reactions were supplemented with 1 mg/ml human fibrinogen and 0.2 mg/ml human IgGs before experimentation. Platelets were stimulated with bacteria and other agonists, including crosslinked collagen-related peptide (CRP-xl), thrombin receptor activator peptide 6 amide (TRAP-6) and ADP. To crosslink FcγRIIA, platelets were incubated with anti-FcγRIIA monoclonal antibody (mAb) (4 μg/ml, clone IV.3) for 2 minutes, followed by the addition of crosslinking F(ab’)_2_ rabbit anti-mouse IgG (30 μg/ml), altogether designated as IV.3-xl hereafter.

### Platelet Factor 4 Secretion

Supernatants from light transmission aggregometry reactions were collected and analyzed for human platelet factor 4 (PF4) by enzyme-linked immunosorbent assay (ELISA) (R&D Systems), as described in **Supplementary Information** (29).

### Platelet Scavenging and Spreading

PRP samples diluted to 2x10^7^ platelets/ml including 15% autologous plasma were incubated for 1 hour with *S. aureus* Newman or fibrinogen immobilized on glass coverslips. Cells were stained with Hoechst 33342 (to visualize bacteria) and TRITC phalloidin (to visualize platelets) and imaged using a Zeiss Axio Observer fluorescence microscope equipped with ApoTome structured illumination and an AxioCam 506 camera (Zeiss, UK). See **Supplementary Information** for details.

### Flow Cytometry

Flow cytometry was performed in PRP samples using a BD LSR Fortessa cell analyzer (BD Biosciences) as described in **Supplementary Information**. Data were analyzed using the FlowJO software (BD Biosciences).

### Phospho-Tec ELISA

WP (10x10^8^/ml) supplemented with 1 mg/ml fibrinogen plus 0.2 mg/ml hIgGs were stimulated as required in aggregation conditions. Samples were lysed 3 minutes after the start of aggregation, or at a parallel time point in the case of samples where inhibitors blocked aggregation, by adding an equal volume of 2x lysis buffer (29) and analyzed using a RayBio human phosphotyrosine Tec ELISA kit (RayBiotech. Georgia, USA) following the manufacturer’s instructions.

### Western Blot

Protein detection in whole platelet lysates was done by SDS-PAGE and near-Infrared Western Blot Detection (LI-COR Biosciences) using lysates collected from PRP samples in the absence of αIIbβ3 inhibitors, as previously described (29) with minor modifications (see **Supplementary Information** for details).

### Statistical Analysis

GraphPad Prism was used to perform the statistical analysis, including normality Kolmogorov-Smirnov and Shapiro-Wilk tests. Data are presented as the mean ± SD. In case of comparisons between two groups, means were evaluated using Student t-test or Mann-Whitney U test, for parametric and non-parametric data, respectively. If more than two groups were considered, one-factor or two-factor analysis of variance (ANOVA) or Kruskal-Wallis test (for parametric and non-parametric data, respectively) followed by multiple comparison tests were performed. For categorical variables, p values were calculated using the chi-square test or Fisher’s exact test. P ≤ 0.05 was considered significant.

## Results

### CLL Patients’ Characteristics

A total of 34 untreated and 32 ibrutinib-treated CLL patients were enrolled with their characteristics summarized in **Table 1**. The median age was 72 years, and most patients received concurrent medications for co-morbidities (**Supplementary Table 1**). The median number of drugs excluding ibrutinib was four for ibrutinib-treated, and three for untreated patients (**Supplementary Table 2**). Antiplatelet and anticoagulants were present in 6% of ibrutinib-treated (e.g., aspirin) and 18% of untreated (e.g., aspirin, clopidogrel, warfarin) CLL patients (**Supplementary Table 1**). Notably, 59% of ibrutinib-treated patients were taking prophylactic antibiotics, and 13% were on combined chemotherapy (venetoclax).

**Table 1:** Characteristics of chronic lymphocytic leukemia patients enrolled in the study.

	Overall (n=66)		Ibrutinib-untreated CLL (n=34)		Ibrutinib-treated CLL (n=32)		P value*
Sex:							
Male	43	(65%)	21	(62%)	22	(69%)	0.61
Female	23	(35%)	13	(38%)	10	(31%)	
Age (years)	72	(45-86)	72.5	(45-86)	71	(55-82)	0.93
17p deletion							
Positive	5	(8%)	0	(0%)	5	(16%)	0.02
Negative	61	(92%)	34	(100%)	27	(84%)	
Trisomy 12							
Positive	0	(0%)	0	(0%)	0	(0%)	>0.99
Negative	66	(100%)	34	(100%)	32	(100%)	
11q deletion							
Positive	7	(11%)	1	(3%)	6	(19%)	0.05^**^
Negative	59	(89%)	33	(97%)	26	(81%)	
13q deletion							
Positive	14	(21%)	0	(0%)	14	(44%)	<0.001 ^**^
Negative	52	(79%)	34	(100%)	18	(56%)	
β2 microglobulin	2.45	(1.4-6.5)	2.3	(1.4-6.5)	2.5	(1.5-6.3)	0.27
IgG at Diagnosis (g/L)	8.9	(3.1-22)	9.15	(3.1-21.5)	8.8	(5.2-22)	0.97
CD38							
Positive	20	(30%)	9	(26%)	11	(34%)	0.59
Negative	46	(70%)	25	(74%)	21	(66%)	
Binet stage^#^							
A	49	(74%)	23	(68%)	26	(81%)	0.12
B	4	(6%)	4	(12%)	0	(0%)	
C	11	(17%)	6	(18%)	5	(16%	
Time from Diagnosis (years)	4	(0.1-23.4)	1.95	(0.1-13.9)	8.1	(1-23.4)	<0.001 ^**^
Time on Ibrutinib (years)	-		-		1.35	(0.1-3.4)	-
Number of Medications excluding ibrutinib	4	(0-9)	3	(0-9)	4	(1-8)	–
Hb (g/L)	133	(88-163)	129	(87-152)	139.5	(95-163)	0.57
WCC (x10^9^/L)	20	(2-379)	38	(3-379)	11.65	(2-319)	0.009 ^**^
Lymphocyte (x10^9^/L)^##^	16	(0.6-372)	40	(1.9-372)	3	(0.6-279)	<0.001 ^**^
Platelet Count (x10^9^/L)	152	(39-378)	166	(39-378)	147	(75-253)	0.05^**^

Hb, hemoglobin; WCC, white blood cell count. ^#^For Binet staging, untreated patients are n=33, and treated patients are n = 31. ^##^For lymphocyte counts, untreated patients were n = 13, and treated patients were n = 16. *Significant difference (p ≤ 0.05).

Data is shown as number (%) or median (range) as appropriate. Cytogenetic tests, CD38 positivity test, and IgG and β2 microglobulin levels are at time of diagnosis; the rest of measurements are at time of blood sample collection. All p values are for comparisons between ibrutinib-untreated and ibrutinib-treated CLL groups. P values for continuous variables were evaluated using Student t-test or Mann-Whitney test, for parametric and non-parametric data, respectively. For categorical variables, p values were calculated using the chi-square test or Fisher’s exact test. P ≤ 0.05 was considered significant. See Supplementary Information for normal hematological and serum immunoglobulin reference ranges, and **Supplementary Tables 1**, **2** for information on patients’ concurrent medications.

### CLL Patients Taking Ibrutinib Have Impaired Platelet Activation in Response to FcγRIIA Crosslinking and Bacteria

Bacteria interact with plasma proteins (including IgGs) and platelet receptors in a bacterial strain-specific manner (34). *S. aureus* Newman and *E. coli* RS218 are known to cause IgG-dependent FcγRIIA-mediated aggregation and granule secretion in platelets derived from healthy donors (29, 31). Therefore, we asked whether platelets from CLL patients would be similarly responsive to bacteria and, if so, whether ibrutinib treatment would interfere with platelet immune functions. We tested platelet activation induced by clustering of FcγRIIA *via* IV.3-xl, bacteria, and GPVI-specific agonist, CRP-xl, in the presence of plasma. We also stimulated platelets with TRAP-6 and ADP, which are agonists for G-protein coupled receptors that are not affected by ibrutinib (12–14).

**Figure 1:**
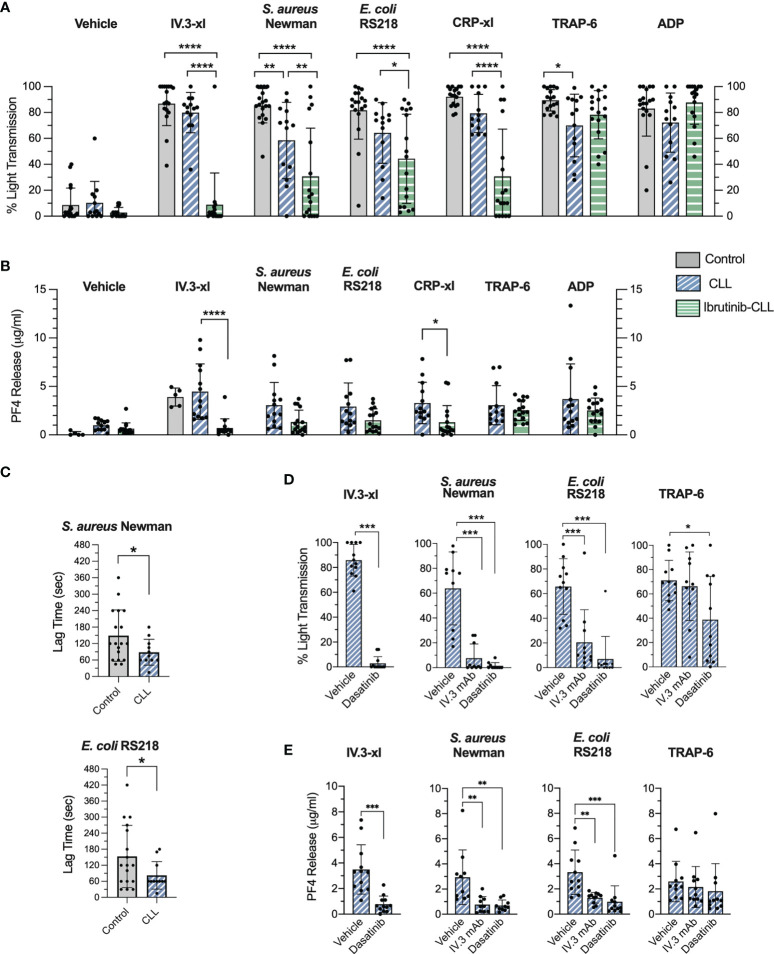
CLL platelet aggregation and secretion in response to bacteria and IV.3 mAb mediated FcγRIIA crosslinking are impaired in CLL patients taking ibrutinib. **(A)** Characterization of platelet aggregation responses in healthy controls (n=17-18), untreated (n=13) and ibrutinib-treated (n=16-17) CLL patients. PRP samples were stimulated with crosslinked IV.3 mAb (IV.3-xl; 4 μg/ml mAb IV.3 followed by 30 μg/ml F(ab’)2 rabbit anti-mouse IgG), *S. aureus* Newman, *E. coli* RS218, 3 μg/ml CRP-xl, 3 μM TRAP-6, or 10 μM ADP. Aggregation was recorded by light transmission aggregometry. Reactions were run for 20 minutes from onset of aggregation for IV.3-xl and bacteria, and 10 minutes for the rest of agonists, and maximum aggregation was calculated. **(B)** Characterization of α-granule secretion in ibrutinib-untreated (n=13) and treated (n=15-16) CLL patients. Supernatants from PRP aggregation reactions were collected at 20 minutes of onset of aggregation for IV.3-xl and bacteria, and 10 minutes in the case of other agonists. Levels of PF4 as a marker of α-granule secretion were measured by PF4 ELISA. **(C)** Lag time for onset of platelet aggregation in response to bacteria. For healthy donor and ibrutinib-untreated platelet samples, the lag time for onset of aggregation (e.g., time between bacteria injection and start of platelet aggregation) was calculated in those cases in which aggregation took place in response to *S. aureus* Newman (healthy controls, n=18; CLL, n=11) and *E. coli* RS218 (healthy controls, n=17; CLL, n=11). **(D)** Effect of FcγRIIA and Src inhibition on ibrutinib-untreated CLL platelet aggregation. PRP samples (n=10-12) were incubated with 4 μM dasatinib (Src inhibitor) for 2 min, 20 μg/ml IV.3 mAb (FcγRIIA inhibitor) for 10 minutes, or vehicle before being stimulated with agonists and monitored by light transmission aggregometry. **(E)** Effect of FcγRIIA and Src inhibition on ibrutinib-untreated CLL platelet α-granule secretion. Supernatants from aggregation reactions above were collected at 20 minutes of onset of aggregation for IV.3-xl and bacteria, and 10 minutes in the case of other agonists. PF4 release was measured by ELISA. Data is shown as mean ± SD. Statistical significance was calculated using two-way ANOVA for **(A, B)**, or one-way ANOVA for **(D, E)**, followed by Tukey’s or Sidak’s multiple comparison correction. An unpaired t-test was performed for **(C)** (*p ≤ 0.05, **p ≤ 0.01, ***p ≤ 0.001, ****p ≤ 0.0001).

Platelets derived from untreated CLL patients responded to FcγRIIA crosslinking (IV.3-xl) and CRP-xl at levels comparable to controls (**Figure 1A**). *S. aureus* Newman and *E. coli* RS218 induced full or nearly full aggregation in 8 and 9 out of 13 samples respectively, similarly to TRAP-6 and ADP, with aggregation to *S. aureus* Newman and TRAP-6 being significantly lower than in controls (**Figure 1A**). The antiplatelet agent, clopidogrel, was taken by two CLL patients, but their platelets aggregated fully to bacteria; however, two aspirin-treated CLL patients showed a reduction in aggregation to bacteria, especially *S. aureus* Newman (**Supplementary Figure 1**).

Platelet aggregation to *S. aureus* Newman and *E. coli* RS218 is characterized by a lag time between bacterial stimulation and start of aggregation that contrasts with the immediate onset of aggregation caused by IV.3-xl (29, 31). Ibrutinib-untreated CLL platelets in which aggregation in response to bacteria was detected (**Figure 1A**) showed a statistically significant decrease in lag times compared to controls (**Figure 1C**). Overall, our data indicates that platelets from ibrutinib-free CLL patients aggregate to bacteria in the presence of autologous plasma, although a trend to decreased aggregation compared to FcγRIIA crosslinking is seen.

In contrast, platelets from ibrutinib-treated CLL patients showed strong inhibition of IV.3-xl-induced aggregation, and a significant decrease in aggregation to bacteria (**Figure 1A**). As opposed to IV.3-xl, heterogeneity in platelet aggregation to CRP-xl was observed in our ibrutinib-treated platelet group (**Figure 1A**). In previous studies, defects in collagen-induced platelet aggregation were detected in only a subset of ibrutinib-treated patients (12, 13); and the presence of ibrutinib low and high sensitivity groups of both healthy individuals and CLL patients regarding platelet responses to collagen has been reported (17). As published (12, 13), responses to TRAP-6 and ADP (which signal through G-protein coupled receptors) were mostly unaffected in our ibrutinib-treated group (**Figure 1A**). Differences in aggregation among the three clinical groups were not due to varying platelet concentrations in PRP samples (**Supplementary Figure 2**).

We next measured release of PF4, a chemokine that can interact with bacteria directly (29, 37), as a marker of α-granule secretion. PF4 was secreted by ibrutinib-untreated CLL platelets upon IV.3-xl and bacteria stimulation (**Figure 1B**) to similar levels as healthy controls (**Figures 1B** and **4C**) (29). In contrast, ibrutinib-treated CLL patients showed an inhibitory trend of PF4 secretion in response to bacteria, IV.3-xl and CRP-xl, with the latter two being significantly reduced compared to untreated CLL platelets (**Figure 1B**). PF4 release in response to TRAP-6 and ADP was not affected by ibrutinib treatment (**Figure 1B**).

To investigate if the CLL platelet responses observed above were mediated by FcγRIIA, aggregation and secretion were measured after incubating PRP with IV.3 mAb, which without the crosslinking F(ab’)_2_ inhibits FcγRIIA activation. Consistent with previous observations in healthy donor platelets (29, 31), CLL platelet aggregation and PF4 secretion to *S. aureus* Newman and *E. coli* RS218 were inhibited by IV.3 mAb and by the Src inhibitor, dasatinib (**Figures 1D, E**).

Thus, our results support that platelet immune recognition of bacteria *via* FcγRIIA is impaired in CLL patients treated with ibrutinib.

### The Ability of Platelets to Scavenge *S. aureus* Newman Is Impaired by Ibrutinib Treatment in CLL Patients

**Figure 2:**
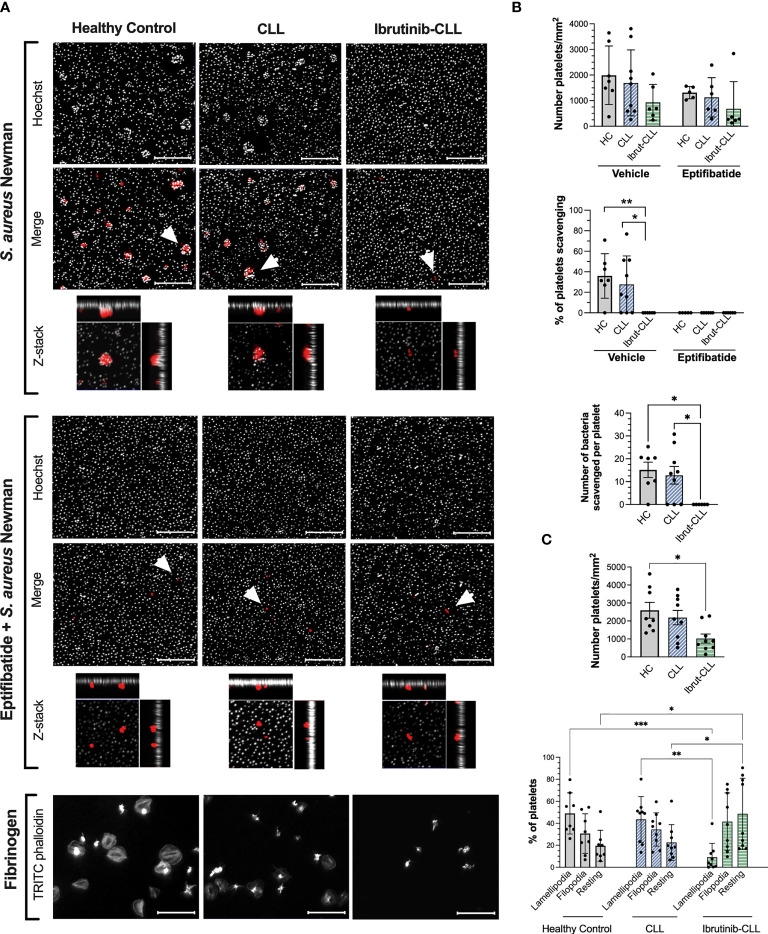
*S. aureus* Newman can be scavenged by platelets from ibrutinib-untreated but not ibrutinib-treated CLL patients. PRP samples from healthy controls, ibrutinib-untreated CLL and ibrutinib-treated CLL were adjusted to 2 x10^7^ platelets/ml and 15% autologous plasma. Samples were incubated with eptifibatide (9 μM) to inhibit integrin αIIbβ3 or vehicle for 2 minutes and incubated for 1 hour with *S. aureus* Newman or fibrinogen immobilized on glass coverslips. Coverslips were then washed, fixed, permeabilized, and stained with Hoechst 33342 (grey color, bacteria staining) and TRITC phalloidin (red color, platelet staining). Coverslips were mounted and visualized by fluorescence microscopy. **(A)** Representative images are presented, with scale bars showing 20 μm. For *S. aureus* Newman, arrowheads indicate examples of clusters of bacteria scavenged by platelets, and maximum projections of Z-stack images of individual platelets are included. **(B)** Platelet interactions with *S. aureus* Newman. Graphs show number of platelets per surface area (top panel), the percentage of platelets scavenging bacteria (middle panel) and the number of bacteria per cluster (bottom panel). Number of samples per clinical group were as follows: healthy controls, n=7 vehicle and n=5 eptifibatide; CLL, n=9 vehicle and n=6 eptifibatide; ibrutinib-treated CLL, n=6 for both vehicle and eptifibatide. **(C)** Platelet spreading on immobilized fibrinogen. Graphs show number of platelets per surface area and percentage of platelets with filopodia (finger-like cellular protrusions) and lamellipodia (laterally extended protrusions characteristic of later stages of spreading). Healthy controls, n=8; CLL, n=9, ibrutinib-CLL, n=9. All data is shown as mean ± SD. Statistical significance was calculated using one-way or two-way ANOVA followed by Tukey’s multiple comparison correction (*p ≤ 0.05, **p ≤ 0.01, ***p ≤ 0.001).

As innate immune effectors, platelets scan the vasculature and collect bacteria (21). Therefore, we next investigated if CLL platelets retain the capacity to scavenge bacteria and whether this is impaired by ibrutinib. Platelets from healthy controls and CLL patients were found to scavenge *S. aureus* Newman in the presence of autologous plasma with an average (± SD) of 15 ± 8.5 and 13 ± 11.5 bacteria per platelet (bacteria clusters), respectively (**Figures 2A, B**). Pre-treatment of PRP samples with the integrin αIIbβ3 inhibitor, eptifibatide, abolished scavenging of bacteria by both control and CLL platelets (**Figures 2A, B**), pointing out to a key role for the integrin in this response. Scavenging was also dependent upon FcγRIIA (**Supplementary Figure 3**). Remarkably, platelets from CLL patients taking ibrutinib could bind to *S. aureus* Newman, but showed no morphological changes indicative of activation as compared to controls and were not able to scavenge bacteria (**Figures 2A, B**).

The same platelet samples were tested for spreading on immobilized fibrinogen. During spreading, the actin cytoskeleton rearranges causing cellular protrusions including finger-like filopodia and laterally extended lamellipodia; the latter being characteristic of later stages of spreading (38). A statistically significant decrease in total number of CLL platelets binding to fibrinogen was detected compared to healthy controls (**Figures 2A, C**), however, both groups showed similar percentages of platelets with lamellipodia. In contrast, platelets from CLL patients taking ibrutinib bound to fibrinogen to similar levels as controls, but did not spread (**Figures 2A, C**), consistent with published studies performed with washed platelets (14, 39). These data show decreased efficiency in platelet bacteria-scavenging ability and spreading in CLL patients treated with ibrutinib.

### Changes in CLL Platelet Granularity and Membrane Expression of αIIbβ3 and GPVI, but not FcγRIIA, Are Differentially Dependent on Ibrutinib Treatment

**Figure 3:**
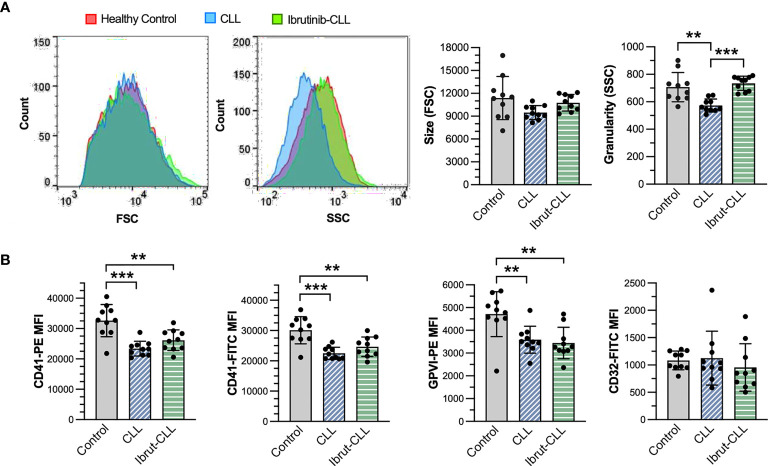
Although different in granularity, platelets from ibrutinib-treated and untreated CLL patients similarly reduce membrane expression of αIIbβ3 and GPVI, but not FcγRIIA. PRP samples from healthy donors (n=10), CLL (n=10) and ibrutinib-treated CLL (n=10) patients were diluted to 1 x10^7^ platelets/ml and incubated with monoclonal antibodies against human integrin αIIb (CD41), GPVI and FcγRIIA (CD32) before being analyzed by flow cytometry. **(A)** Left histograms show size and granularity in gated platelets, respectively as forward (FSC) and side (SSC) scatter, in representative samples. The bar graphs (right) show the distribution of platelet size and granularity across the three experimental groups. **(B)** Bar graphs show the mean fluorescence intensity (MFI) of CD41, GPVI, and CD32 in gated platelets, across the three experimental groups. For CD41, technical replicates with two different antibody clones (PE-conjugated clone 5B12, FITC-conjugated clone P2) are shown (left panels). Statistical significance was evaluated in ANOVA analyses, followed by Tukey’s multiple comparison correction. Data is presented as mean ± SD. (**p ≤ 0.01, ***p ≤ 0.001).

The reduction in platelet responses to bacteria observed in ibrutinib-treated CLL patients could be due to heterogeneity in surface expression of important receptors like FcγRIIA and αIIbβ3 (40). Therefore, we performed flow cytometry analyses of platelets derived from our three clinical groups to measure membrane protein levels of αIIbβ3, FcγRIIA and GPVI in PRP samples (**Supplementary Figure 4**). This analysis revealed no change in cell size, but a significant decrease in granularity/complexity in untreated CLL platelets (**Figure 3A**). We also observed significant reductions of αIIbβ3 (as detected by two different mAb clones against CD41, the αIIb subunit) and GPVI, but not FcγRIIA (CD32), in CLL platelets independently of ibrutinib treatment when compared to healthy control platelets (**Figure 3B**). This suggests that decreased levels of αIIbβ3 in blood circulating platelets are unlikely to be the major or only cause of inhibition of ibrutinib-treated CLL platelet responses to bacteria.

### Ibrutinib and Acalabrutinib Inhibit Healthy Donor Platelet Activation in Response to Antibody-Mediated FcγRIIA Crosslinking and Bacteria

**Figure 4:**
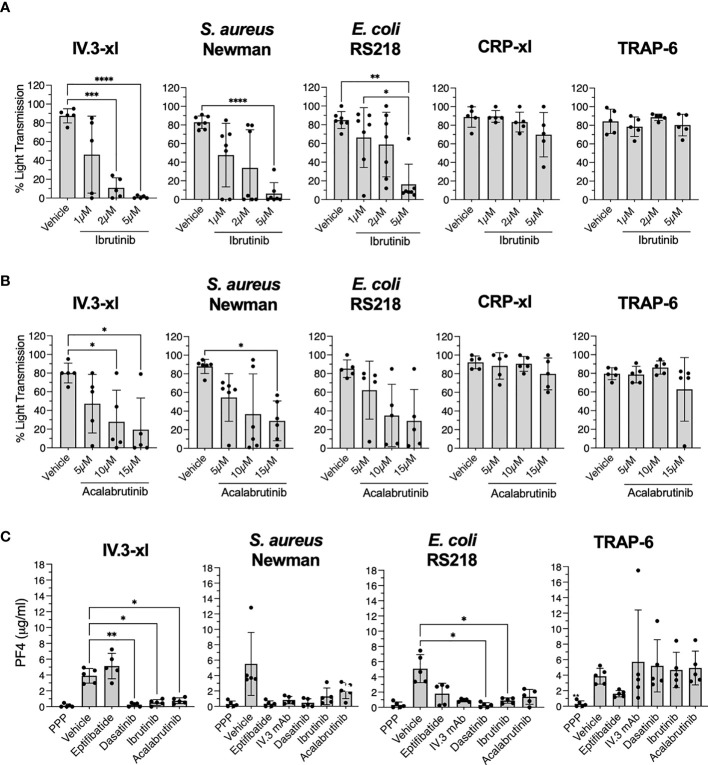
Ibrutinib and acalabrutinib inhibit healthy control platelet activation by bacteria and IV.3 mAb mediated FcγRIIA crosslinking in the presence of plasma. **(A)** Characterization of the effect of ibrutinib on platelet aggregation. PRP from healthy controls was incubated *in vitro* with different doses of ibrutinib for 5 minutes before stimulation with the following agonists: crosslinked IV.3 mAb (IV.3-xl; 4 μg/ml IV.3 mAb followed by 30 μg/ml F(ab’)2 rabbit anti-mouse IgG; n=5), *S. aureus* Newman (n=7), *E. coli* RS218 (n=7), 3 μg/ml CRP-xl (n=5) and 3 μM TRAP-6 (n=5). Aggregation was measured by light transmission aggregometry. Reactions were run for 20 minutes from onset of aggregation for IV.3-xl and bacteria, and 10 minutes for the rest of agonists, and maximum aggregation was calculated. **(B)** Characterization of the effect of acalabrutinib on platelet aggregation. PRP from healthy controls was incubated *in vitro* with different doses of acalabrutinib for 5 min before stimulating with agonists: IV.3-xl (n=5), *S. aureus* Newman (n=6), *E. coli* RS218 (n=5), 3 μg/ml CRP-xl (n=5) and 3 μM TRAP-6 (n=5). Aggregation was measured by light transmission aggregometry as above. **(C)** PRP from healthy controls (n=5) was incubated with vehicle or inhibitors, 9 μM eptifibatide (αIIbβ3 inhibitor), 20 μg/ml IV.3 mAb (to inhibit FcγRIIA), 4 μM dasatinib (Src inhibitor), or iBtks, 5 μM ibrutinib and 15 μM acalabrutinib. Samples were then stimulated with stated agonists. Supernatants from aggregation reactions were collected at 20 minutes of onset of aggregation for IV.3-xl and bacteria, and 10 minutes for TRAP-6. Levels of PF4 as a marker of α-granule secretion were measured by PF4 ELISA. Data is shown as mean ± SD. Statistical significance was calculated using one-way ANOVA followed by Tukey’s multiple comparison correction (*p ≤ 0.05, **p ≤ 0.01, ***p ≤ 0.001, ****p ≤ 0.0001).

To investigate the direct effect of ibrutinib on platelet response to bacteria, we performed light transmission aggregometry with healthy control PRP stimulated *in vitro* with different concentrations of ibrutinib (**Figure 4A** and **Supplementary Figure 5A**). Pre-incubation with 5µM ibrutinib for 5 minutes significantly inhibited platelet aggregation to *S. aureus* Newman and *E. coli* RS218, but did not cause a significant reduction of aggregation in response to CRP-xl or TRAP-6. A lower dose of ibrutinib (2 µM) was able to abolish aggregation to FcγRIIA crosslinking (IV.3-xl) (**Figure 4A**). Longer pre-incubation times with ibrutinib (20 and 40 minutes) did not significantly reduce the concentration of drug needed to inhibit aggregation to bacteria (data not shown). As internal controls, IV.3 mAb alone (FcγRIIA inhibitor) and dasatinib (Src inhibitor) were used in PRP, and both inhibited aggregation to *S. aureus* Newman and *E. coli* RS218, but not TRAP-6 (**Supplementary Figure 5C**), as previously reported (29, 31). The integrin αIIbβ3 inhibitor eptifibatide, as expected, blocked aggregation in response to all agonists including TRAP-6 (**Supplementary Figure 5C**) (29, 31).

The high concentrations of ibrutinib needed to inhibit aggregation in PRP contrasted with previous studies done in washed platelets (12, 14, 16), thus we wondered if binding of ibrutinib to plasma proteins (41) may explain the difference. As seen in **Supplementary Figure 6A**, washed platelet aggregation in response to *S. aureus* Newman was inhibited by 0.5 µM ibrutinib, while 0.1 µM ibrutinib was sufficient to inhibit the response to *E. coli* RS218 and FcγRIIA crosslinking (IV.3-xl). A higher dose of ibrutinib (1 µM) was necessary to inhibit washed platelet aggregation stimulated with CRP-xl. None of the ibrutinib concentrations tested in PRP and washed platelets caused inhibition of TRAP-6 induced aggregation (**Figure 4A** and **Supplementary Figures 5A, 6A**).

The second generation iBtk, acalabrutinib, was also assessed for its ability to inhibit platelet responses to bacteria. Like ibrutinib, acalabrutinib binds irreversibly to Btk at C481 and is highly bound to plasma proteins (42, 43). In PRP, 10-15 µM acalabrutinib impaired aggregation after IV.3-xl and bacteria exposure, but not in response to CRP-xl and TRAP-6 (**Figure 4B** and **Supplementary Figure 5B**). In washed platelets, the inhibitory effects of acalabrutinib were more prominent. Aggregation to IV.3-xl was inhibited by 5-10 µM acalabrutinib, while for bacteria, 1µM and 10 µM inhibited aggregation upon exposure to *E. coli* RS218 and *S. aureus* Newman respectively (**Supplementary Figure 6B**). Washed platelet aggregation after stimulation with TRAP-6 and CRP-xl was unaffected or only partially inhibited (10 µM) by acalabrutinib respectively (**Supplementary Figure 6B**), as previously described (17, 44).

Ibrutinib and acalabrutinib also inhibited platelet α-granule secretion (PF4) in response to IV.3-xl and bacteria, but not to TRAP-6 (**Figure 4C**). As previously reported (29, 31), eptifibatide blocked PF4 release in response to bacteria, but not to IV.3-xl (**Figure 4C**). Moreover, PF4 release in response to bacteria (but not TRAP-6) was inhibited by blocking FcγRIIA with IV.3 mAb alone (**Figure 4C**). This confirms previous observations of the interplay between FcγRIIA and αIIbβ3 in mediating platelet activation, including secretion, to bacteria.

Hence, in keeping with the above results from our clinical cohort of ibrutinib-treated CLL patients, iBtks strongly and directly inhibit immune function even in platelets derived from healthy donors.

### Bacteria Induce FcγRIIA- and αIIbβ3-Dependent Btk Activation That Is Inhibited by iBtks

**Figure 5:**
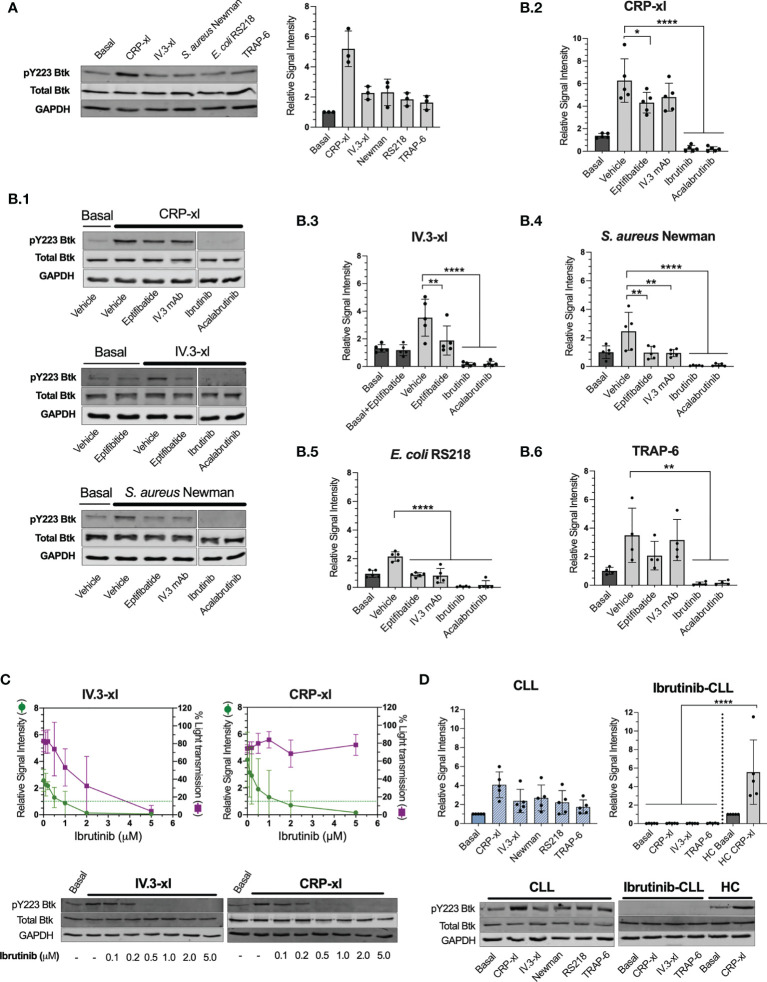
Platelet stimulation by bacteria or crosslinked IV.3 mAb causes Btk activation in healthy donors and CLL patients that is inhibited by ibrutinib and acalabrutinib. Phosphorylation of Btk at Y223 was measured by immunoblotting in whole cell lysates collected from healthy control or CLL PRP aggregation reactions using the following agonists: 3 μg/ml CRP-xl, crosslinked IV.3 (IV.3-xl; 4 μg/ml mAb IV.3 followed by 30 μg/ml F(ab’)2 rabbit anti-mouse IgG), *S. aureus* Newman, *E. coli* RS218, and 3 μM TRAP-6. In some cases, platelet inhibitors were used as indicated below. All lysates were collected at 3 minutes after the start of aggregation, or a parallel time point in samples in which aggregation was inhibited. Lysates were adjusted to an equivalent of 5 x10^8^ platelets/ml as explained in Materials and Methods. **(A)** Btk pY223 phosphorylation in healthy donor platelets stimulated with assorted agonists. Pooled data from three independent experiments done with different donors is shown on the right-hand side graph as mean ± SD. Data was normalized to GAPDH. On the left, one representative experiment is presented. **(B)** Effect of platelet inhibitors on Btk pY223 phosphorylation in healthy control platelets. Before adding agonists, PRP was pre-incubated with either vehicle, 9μM eptifibatide (αIIbβ3 inhibitor, 2 minutes), 20 μg/ml IV.3 mAb (to inhibit FcγRIIA, 10 minutes), or iBtks (5 minutes), 5μM ibrutinib and 15μM acalabrutinib. B.1. Representative images of five independent experiments for CRP-xl, IV.3-xl and *S. aureus* Newman. B.2 to B.6. Pooled data shown as mean ± SD. Five independent experiments were done for all agonists except for TRAP-6 that was tested four times. Data was normalized to GAPDH. Statistical significance was calculated using one-way ANOVA followed by Tukey’s multiple comparison correction. **(C)** Ibrutinib dose-response curve for inhibition of Btk Y223 phosphorylation (green) and aggregation (purple) in response to IV.3-xl (n=5) and CRP-xl (n=4). Representative results are shown below the graphs. **(D)** Btk Y223 phosphorylation in PRP from ibrutinib-untreated (n=5) and treated (n=5) CLL patients stimulated with assorted agonists. Healthy control (HC) samples were included as positive controls. Statistical significance was calculated using one-way or two-way ANOVA followed by Tukey’s multiple comparison correction (*p ≤ 0.05, **p ≤ 0.01, ****p ≤ 0.0001).

We showed that ibrutinib can inhibit platelet immune recognition of bacteria, mediated by FcγRIIA, whilst not impacting on the surface levels of this receptor, thus we sought to investigate downstream intracellular signaling events. The binding of ibrutinib and acalabrutinib to Btk C481 blocks Btk autophosphorylation in Y223 that is critical for its activation (5). FcγRIIA crosslinking with non-physiological agonists induces Btk Y223 phosphorylation (35), yet it remains unknown if this is also critical downstream of bacteria recognition. In healthy donors, phosphorylation of Btk Y223 was detected in PRP after IV.3-xl and bacteria exposure, although at lower levels than those induced by CRP-xl (**Figure 5A**). For *S. aureus* Newman and *E. coli* RS218, inhibition of FcγRIIA (with IV.3 mAb alone) and αIIbβ3 (with eptifibatide) reduced Btk phospho-Y223 signal to basal levels (**Figures 5B.1, B.4** and **B.5**), which indicates that both receptors control Btk activation in response to bacteria, as opposed to CRP-xl (**Figures 5B.1, B.2**) and TRAP-6 (**Figure 5B.6**). For all agonists tested, Btk phospho-Y223 was inhibited below basal levels by ibrutinib and acalabrutinib as expected (**Figures 5A, B**).

We wondered whether signaling through FcγRIIA, rather than GPVI, would be more reliant on Btk activity, and hence sensitive to ibrutinib inhibition. Therefore, we analyzed if there was a correlation between the ibrutinib concentration found to inhibit Btk Y223 phosphorylation and that needed to inhibit aggregation. Upon IV.3-xl, concentrations of ibrutinib that reduced Btk phospho-Y223 to basal levels (1 μM) or below (2 μM) were also able to partially decrease aggregation (**Figure 5C**), with total inhibition of aggregation requiring higher concentrations of ibrutinib (5 μM). In contrast, inhibition of Btk Y223 phosphorylation did not correlate with suppression of CRP-xl induced aggregation (**Figure 5C**), as previously reported (16). Thus, phosphorylation of Btk Y223 downstream of bacteria recognition uses both FcγRIIA and integrin pathways, with FcγRIIA potentially being more sensitive to ibrutinib treatment than GPVI, in healthy donor-derived platelets.

We next sought to investigate CLL samples and found that CLL platelets activated Btk in response to IV.3-xl, bacteria, CRP-xl and TRAP-6 to a similar degree as healthy controls (compare **Figures 5A, D**). This contrasted with ibrutinib-treated CLL platelets in which no phosphorylation of Btk Y223 was detected in response to IV.3-xl, CRP-xl and TRAP-6 (**Figure 5D**).

### Bacteria Induce Platelet Tec Kinase Activation That Is Inhibited by Ibrutinib

Platelets express two members of the Tec family of tyrosine kinases, Btk and Tec. Although Btk has an approximate 8 to 10-fold greater level of expression in human platelets (45), studies suggest that ibrutinib-mediated inhibition of Tec has important consequences in platelet function (14, 16). Thus, we aimed to decipher if Tec activation takes place downstream of FcγRIIA-mediated platelet response to bacteria as seen for IV.3-xl (35). Due to the lack of phospho-specific antibodies to detect Tec autophosphorylation site Y206 (46), total levels of Tec tyrosine phosphorylation were measured by ELISA.

**Figure 6:**
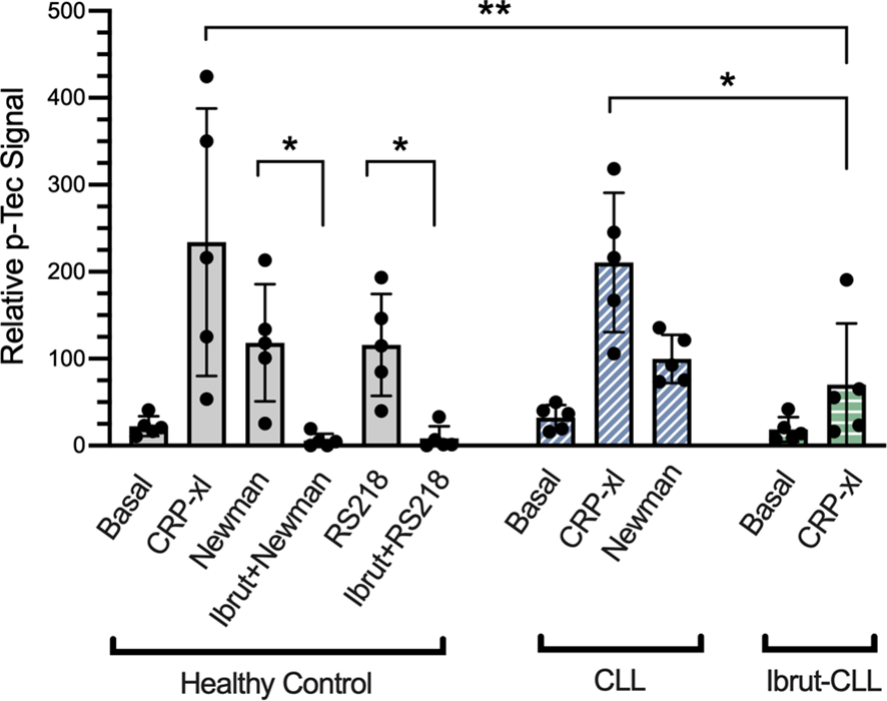
Platelet responses to bacteria involve Tec kinase activation. Characterisation of platelet Tec phosphorylation in healthy controls (n=5), ibrutinib-untreated CLL (n=5) and ibrutinib-treated CLL (n=5) patients. Washed platelets from the three clinical groups were supplemented with fibrinogen and human IgG (commercially available IgGs pooled from healthy donors). Healthy control platelets were incubated *in vitro* with 5 μM ibrutinib, 15 μM acalabrutinib or vehicle for 5 minutes before adding agonists. Platelet samples were stimulated with either 3 μg/ml CRP-xl, *S. aureus* Newman or *E. coli* RS218. Relative phosphorylation of Tec was measured by a phospho-Tec ELISA. Data is shown as mean ± SD. Two-way ANOVA and Sidak’s multiple comparison test were used to compare CRP-xl responses across the three clinical groups. For the effect of ibrutinib on bacteria-induced phospho-Tec in healthy controls, one way ANOVA followed by Tukey’s multiple comparison correction was done. (*p ≤ 0.05, **p ≤ 0.01).

In healthy donor-derived platelets, Tec phosphorylation increased upon exposure to *S. aureus* Newman and *E. coli* RS218, and was inhibited by pre-treatment with ibrutinib (**Figure 6**). Moreover, when CLL platelets were stimulated with *S. aureus* Newman, Tec phosphorylation reached similar levels as those observed in healthy controls (**Figure 6**). We used CRP-xl to compare Tec phosphorylation across our three clinical groups. As seen in **Figure 6**, healthy donor and untreated CLL platelets stimulated with CRP-xl showed an increase in phospho-Tec that was significantly decreased in platelets from ibrutinib-treated patients. Overall, these data suggest that ibrutinib, both at concentrations used *in vitro* and at therapeutic doses, affects platelet Tec activation.

### XLA-Derived Platelets Respond to Bacteria in an FcγRIIA-Dependent Manner

**Figure 7:**
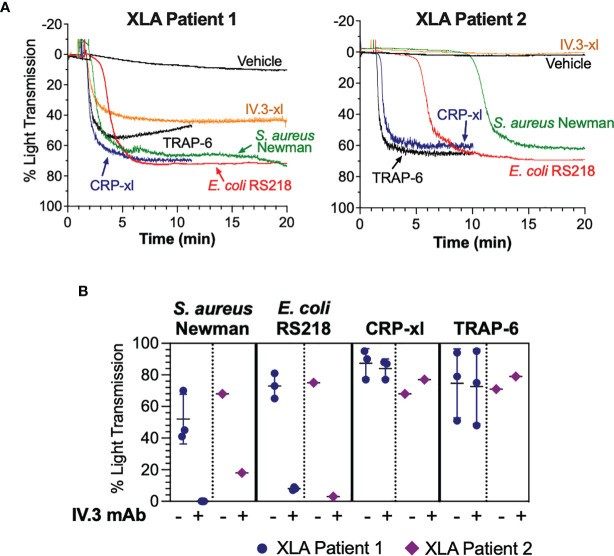
Platelet responses to bacteria can take place in the absence of functional Btk. **(A)** Characterization of bacteria-induced aggregation of platelets from X-linked agammaglobulinemia patients. PRP samples from two XLA patients were stimulated with crosslinked IV.3 mAb (IV.3-xl; 4 μg/ml IV.3 mAb followed by 30μg/ml F(ab’)_2_ rabbit anti-mouse IgG), *S. aureus* Newman, *E*. *coli* RS218, 3 μg/ml CRP-xl, and 3 μM TRAP-6. Aggregation was measured by light transmission aggregometry. **(B)** PRP from XLA patients was incubated with 20 μg/ml IV.3 mAb (to inhibit FcγRIIA) or vehicle, before stimulation with either *S. aureus* Newman, *E. coli* RS218, 3 μg/ml CRP-xl or 3 μM TRAP-6. Aggregation was measured by light transmission aggregometry. Patient 1 (n=3, in different days) had a missense mutation in codon 28 affecting the Btk pleckstrin homology domain, which causes lack of Btk function. Patient 2 (n=1) had a mutation in exon 3 resulting in absence of Btk expression.

Mutations in the *Btk* gene resulting in lack of Btk expression or function cause XLA, a condition characterized by markedly reduced or absent B cells and profound hypogammaglobulinemia (47). To investigate if Btk function is essential for FcγRIIA-mediated platelet responses to bacteria, we obtained PRP from two XLA patients receiving periodic intravenous immunoglobulin treatment. Platelets from both patients aggregated to *S. aureus* Newman and *E. coli* RS218 although with varying lag times (**Figure 7A**). Upon crosslinking of FcγRIIA (IV.3-xl), no or less aggregation was observed in patients 2 and 1 respectively (**Figure 7A**), suggesting that this pathway is more strongly affected by Btk deficiency than bacteria-induced activation. Importantly, XLA platelet responses to bacteria were still dependent on FcγRIIA, as aggregation was blocked by pre-incubating PRP with inhibitory IV.3 mAb alone (**Figure 7B**). Conversely, we detected normal XLA platelet aggregation in response to CRP-xl and TRAP-6 (**Figure 7A**), which was independent of FcγRIIA (**Figure 7B**). These results suggest that while Btk has an active role in FcγRIIA-mediated platelet function, this is not essential for FcγRIIA-mediated responses to bacteria.

## Discussion

This study has focused on platelet immune responses in CLL in the context of bacterial infection and ibrutinib therapy. Infectious complications are a major burden in CLL, and the increased risk of infection is multifactorial, including immunosuppression inherent to disease, patients’ age and co-morbidities, and therapy toxicity (3, 4, 6, 27, 28). In CLL, alterations can be found in both the adaptive and innate immune systems, namely T lymphocyte dysfunction, hypogammaglobulinemia, complement defects, and impairment in antimicrobial activity of neutrophils and monocytes (4, 25, 26). Because it is possible that CLL platelets had immune function abnormalities that could be exacerbated by ibrutinib, the first part of our study had two aims: to characterize platelet responses to bacteria in CLL (e.g., in the absence of CLL-targeted treatment) and to investigate the effect of ibrutinib therapy on these responses.

Overall, ibrutinib-untreated CLL platelets responded to *S. aureus* and *E. coli* in an FcγRIIA-dependent manner in the presence of autologous plasma, as seen by platelet aggregation, secretion and scavenging of bacteria. We found that platelet scavenging of *S. aureus* Newman was both FcγRIIA and αIIbβ3-dependent. However, more investigations are needed to further characterize the molecular mechanisms underlying platelet scavenging of pathogens, including potential bacterial-strain specific processes. Previous studies suggest that alternative mechanisms could take place. Gaertner et al. (21) have shown that platelets can scavenge fibrin-bound *E. coli* and *S. aureus*, both *in vitro* and *in vivo*, boosting the phagocytic activity of neutrophils; while Palankar et al. (22) described a mechanism by which PF4 binding to *E. coli* promotes antibody opsonization and FcγRIIA-mediated platelet spreading that covers the opsonized bacteria leading to bacterial killing (22). Platelets have also been shown to kill *S. aureus* Newman, but not *S. pneumoniae*, in a process that was FcγRIIA independent (23). Moreover, the releasate derived from platelet aggregation can directly kill *S. aureus* and enhance the antimicrobial function of macrophages (23, 48).

Despite the overall response to bacteria, some variation was observed in ibrutinib-untreated CLL platelets, with some samples not reaching full activation. Differences in bacteria-induced aggregation in healthy donor platelets are not uncommon (49, 50), however, variation in CLL could be due to additional factors like co-morbidities and related medications (51). Indeed, most of our CLL patients were taking multiple medications reflecting a typical population of CLL patients often with multiple comorbidities. The increased bleeding risk found in ibrutinib-treated patients is exacerbated by concurrent intake of antiplatelet or anticoagulant therapy (7), and *in vitro* studies show that ibrutinib amplifies the effect of cangrelor and indomethacin on platelets (14, 17). However, in our study a minority of patients were taking antiplatelet medications, and thus, future research should be done to investigate the effect of different drug combinations, including iBtk combinations with antiplatelet drugs, on platelet immune functions. Other factors that might explain why platelets from some CLL patients did not activate fully to bacteria are plasma immunoglobulin concentrations (52) and polymorphic variants of FcγRIIA (53, 54). Notably, some of these factors might also account for variable platelet responses to bacteria within healthy individuals (49, 50). Although variations in the expression of FcγRIIA (55) could also affect platelet activation by microorganisms, no significant changes in FcγRIIA levels were detected among our clinical groups.

Three observations suggested alterations in circulating CLL platelets: a decrease in platelet granularity/complexity, an ibrutinib-independent decrease in membrane levels of αIIbβ3 and GPVI compared to controls, and a reduction in lag time for onset of platelet aggregation in response to bacteria; although the latter could also be due to changes in plasma composition in CLL. Our flow cytometry results differ from others in which αIIbβ3 surface expression in CLL platelets was similar to healthy donors (40, 56) but decreased upon ibrutinib treatment (40). Moreover, reduced GPVI levels in CLL platelets were found in one study (56), but not another (40). Platelet alterations could originate during megakaryopoiesis as CLL is characterized by bone marrow dysfunction (57); however, they could also reflect interactions with unknown-disease-related factors once platelets are released into the circulation (57). The modulation of membrane receptor levels can affect platelet reactivity to external stimuli as well as platelet senescence and lifespan (58); and changes in platelet granularity might reflect some degree of activation (with granule centralization) (19). Therefore, future studies are needed to characterize CLL platelet proteomic and structural changes and their potential consequences in platelet function in the presence and absence of iBtks.

A key finding of this investigation is that therapeutic doses of ibrutinib impair platelet responses to *S. aureus* Newman and *E. coli* RS218. While it is still possible that an underlying CLL platelet immune dysfunction is significantly worsened by iBtks, the fact that ibrutinib and acalabrutinib also inhibited platelets derived from healthy donors points out to the ability of iBtks to directly interfere with platelet immune functions (independently of additional CLL-specific changes). Despite some conflicting reports regarding the increased risk of infection during ibrutinib therapy in B-cell malignancies, Ball et al. have recently published a systematic review and meta-analysis of randomized controlled trials, including more than 2000 patients, in which a significantly higher risk of infections associated with ibrutinib treatment has been found (6). Ibrutinib inhibition of platelet immune functions might contribute to infection by interfering with the ability of platelets to contain and kill pathogens and crosstalk with other immune cells (19–24, 48, 59); however, this hypothesis will need further evaluation in view of the complex multifactorial nature of infection complications in CLL. For example, ibrutinib affects neutrophil, monocyte and macrophage responses to fungi (10, 11), and these effects might also have important consequences in responses to bacteria. Furthermore, infections cannot be explained by Btk inhibition alone, as seen by the decline in the risk of infection and neutropenia rate over time on ibrutinib therapy presumably due to gradual immune reconstitution (60–62). Notably, in our study some platelet samples from ibrutinib-treated patients aggregated to bacteria, mainly *E. coli* RS218, suggesting the presence of Btk-independent mechanisms of activation. It is important to point out that immune responses in CLL, including those ones in which platelets might be involved, are likely microorganism dependent. Thus, frequent pathogens like *S. pneumoniae*, *H. influenzae*, but also fungi (63) should be examined in this context.

To clarify if newer iBtks with improved selectivity for Btk over other kinases (5, 42, 64) might be beneficial to treat B-cell malignancies, it is important to understand how Btk and structurally related kinases like Tec work in non-malignant cells, including platelets. For this reason, the second part of our study investigated the role of Btk in platelet immunity. Previous studies on healthy donor platelets reported a role for Btk and Tec in FcγRIIA-mediated platelet activation in response to artificial antibody crosslinking and sera from heparin-induced thrombocytopenia patients (35, 36). Here, we studied Btk and Tec activation downstream of bacteria-stimulated platelets from healthy donors and CLL patients. Bacteria and FcγRIIA crosslinking with IV.3 mAb caused Btk activation, and platelet aggregation that was more sensitive to ibrutinib than CRP-xl stimulation, suggesting that Btk has a role downstream of FcγRIIA, as supported by investigations by Goldmann et al. (36). Although the peak plasma concentration of ibrutinib is only 0.3-0.4 μM (65), Btk activation was abolished in platelets derived from ibrutinib-treated patients, which confirmed that therapeutic doses of ibrutinib target platelet Btk. Nonetheless, bacteria also caused platelet Tec phosphorylation that was inhibited by ibrutinib.

Because platelets are anucleated, intracellular signaling studies often rely on animal models. For GPVI, the relative contribution of Btk and Tec in GPVI-mediated platelet activation was studied using platelets from knockout mice, and only those from double Btk and Tec knockout animals were insensitive to CRP-xl (66). However, this strategy is not feasible in the context of our study because murine platelets do not express FcγRIIA, and platelets from FcγRIIA-transgenic mice do not respond to many bacterial strains that are known to activate human platelets, even when supplemented with human IgGs (29). This should be taken as a warning of the limitations of using animal models that lack FcγRIIA to address platelet immune functions that depend on IgGs, thus linking innate and acquired immune responses. Instead, we tested platelets from two XLA patients, which lack functional Btk, and found that they responded to bacteria in an FcγRIIA-dependent manner, although we detected some impairment in aggregation to FcγRIIA crosslinking (IV.3-xl) that will require further investigation. Most commonly, XLA patients suffer from respiratory infections caused by *S. pneumoniae* and *H. influenza*. Although *S. aureus* infections are also reported (67, 68), generally they are not considered significant problems in patients who receive regular immunoglobulin replacement therapy (47), which further suggests that Btk is not essential for the immune system to control infection by specific bacterial species in the presence of normal IgGs. Altogether, and as found for GPVI (15, 35, 66, 69), our results suggest that either Btk is dispensable for platelet aggregation to *S. aureus* and *E. coli*, or there is enough Btk/Tec kinase functional redundancy to rescue the activating signaling when Btk is absent. It is also possible that other tyrosine kinases can compensate for the loss of Btk, but still be off-targeted by ibrutinib in CLL patients. Platelet-bacteria interactions are complex and can involve multiple receptor-ligand combinations that are bacterial-strain specific (70), therefore, it is also possible that crosstalk between FcγRIIA/αIIbβ3 and other receptors (e.g., GPIb, complement receptor gC1q-R, and Toll-like receptor 2) (33, 34) and signaling pathways can overcome the absence of Btk.

In summary, this study shows that CLL platelets can respond to *S. aureus* and *E. coli* bacteria in an FcγRIIA-dependent manner and provides evidence that ibrutinib impairs such responses. Mechanistically, while FcγRIIA expression was not altered by the treatment, our data demonstrates that ibrutinib inhibits both Btk and Tec kinases downstream of FcγRIIA/αIIbβ3 activation in response to bacteria stimulation. This points out to the importance of evaluating the effect of iBtks used to treat CLL and other hematological malignancies on platelet immune functions.

## Data Availability Statement

The raw data supporting the conclusions of this article will be made available by the authors, without undue reservation.

## Ethics Statement

The studies involving human participants were reviewed and approved by Hull York Medical School Ethics Committee (Ethics reference number 1501) and UK National Health Service Research Ethics Committee (Ethics reference number 08/H1304/35). The patients/participants provided their written informed consent to participate in this study.

## Author Contributions

LN-A designed research, performed most experiments, analyzed data, and wrote the manuscript. AC designed, performed, and analyzed scavenging experiments. ZB and JJ performed experiments. SC designed and supervised flow cytometry research and analyzed data. SK provided access to XLA patients. SH provided key reagents and intellectual input. FR designed and performed platelet scavenging research and analyzed data. DA provided and coordinated access to CLL patients and designed and supervised the study. MA designed and directed research, performed experiments, analyzed data and wrote the manuscript. All authors contributed to the article and approved the submitted version.

## Funding

This work was supported by the University of Hull and Hull University Teaching Hospital Trust. LN-A and ARC were recipients of University of Hull PhD scholarships.

## Conflict of Interest

DA has received honoraria for speaking from Gilead Pharmaceuticals, funding for conference attendance from Bayer and CSL Behring and research funding from Roche.

The remaining authors declare that the research was conducted in the absence of any commercial or financial relationships that could be construed as a potential conflict of interest.

## Publisher’s Note

All claims expressed in this article are solely those of the authors and do not necessarily represent those of their affiliated organizations, or those of the publisher, the editors and the reviewers. Any product that may be evaluated in this article, or claim that may be made by its manufacturer, is not guaranteed or endorsed by the publisher.

## References

[1] HMRN. Haematological Malignancy Research Network (2018). Available at: https://www.hmrn.org/.

[2] Nosari A. Infectious Complications in Chronic Lymphocytic Leukemia. Mediterr J Hematol Infect Dis (2012) 4:2012070. doi: 10.4084/mjhid.2012.070 PMC3507529 23205258

[3] Korona-Glowniak I Grywalska E Grzegorczyk A Roliński J Glowniak A Malm A. Bacterial Colonization in Patients With Chronic Lymphocytic Leukemia and Factors Associated With Infections and Colonization. J Clin Med (2019) 8:861. doi: 10.3390/jcm8060861 PMC6616586 31208150

[4] Teh BW Tam CS Handunnetti S Worth LJ Slavin MA. Infections in Patients With Chronic Lymphocytic Leukaemia: Mitigating Risk in the Era of Targeted Therapies. Blood Rev (2018) 32:499–507. doi: 10.1016/j.blre.2018.04.007 29709246

[5] Wen T Wang J Shi Y Qian H Liu P. Inhibitors Targeting Bruton’s Tyrosine Kinase in Cancers: Drug Development Advances. Leukemia (2021) 35:312–32. doi: 10.1038/s41375-020-01072-6 PMC7862069 33122850

[6] Ball S Das A Vutthikraivit W Edwards PJ Hardwicke F Short NJ. Risk of Infection Associated With Ibrutinib in Patients With B-Cell Malignancies: A Systematic Review and Meta-Analysis of Randomized Controlled Trials. Clin Lymphoma Myeloma Leukemia (2020) 20:87–97.e5. doi: 10.1016/j.clml.2019.10.004 31787589

[7] Lipsky AH Farooqui MZH Tian X Martyr S Cullinane AM Nghiem K. Incidence and Risk Factors of Bleeding-Related Adverse Events in Patients With Chronic Lymphocytic Leukemia Treated With Ibrutinib. Haematologica (2015) 100:1571–8. doi: 10.3324/haematol.2015.126672 PMC4666333 26430171

[8] Munir T Brown JR O’Brien S Barrientos JC Barr PM Reddy NM. Final Analysis From RESONATE: Up to Six Years of Follow-Up on Ibrutinib in Patients With Previously Treated Chronic Lymphocytic Leukemia or Small Lymphocytic Lymphoma. Am J Hematol (2019) 94:1353–63. doi: 10.1002/ajh.25638 PMC6899718 31512258

[9] Honigberg LA Smith AM Sirisawad M Verner E Loury D Chang B. The Bruton Tyrosine Kinase Inhibitor PCI-32765 Blocks B-Cell Activation and is Efficacious in Models of Autoimmune Disease and B-Cell Malignancy. Proc Natl Acad Sci (2010) 107:13075–80. doi: 10.1073/pnas.1004594107 PMC2919935 20615965

[10] Fiorcari S Maffei R Vallerini D Scarfò L Barozzi P Maccaferri M. BTK Inhibition Impairs the Innate Response Against Fungal Infection in Patients With Chronic Lymphocytic Leukemia. Front Immunol (2020) 11:2158. doi: 10.3389/fimmu.2020.02158 32983178 PMC7485008

[11] Blez D Blaize M Soussain C Boissonnas A Meghraoui-Kheddar A Menezes N. Ibrutinib Induces Multiple Functional Defects in the Neutrophil Response Against Aspergillus Fumigatus. Haematologica (2019) 105(2):478–89. doi: 10.3324/haematol.2019.219220 PMC7012467 31171644

[12] Levade M David E Garcia C Laurent P-A Cadot S Michallet A-S. Ibrutinib Treatment Affects Collagen and Von Willebrand Factor-Dependent Platelet Functions. Blood (2014) 124:3991–5. doi: 10.1182/blood-2014-06-583294 25305202

[13] Kamel S Horton L Ysebaert L Levade M Burbury K Tan S. Ibrutinib Inhibits Collagen-Mediated But Not ADP-Mediated Platelet Aggregation. Leukemia (2015) 29:783–7. doi: 10.1038/leu.2014.247 25138588

[14] Bye AP Unsworth AJ Vaiyapuri S Stainer AR Fry MJ Gibbins JM. Ibrutinib Inhibits Platelet Integrin αiibβ3 Outside-In Signaling and Thrombus Stability But Not Adhesion to Collagen. Arterioscler Thromb Vasc Biol (2015) 35:2326–35. doi: 10.1161/atvbaha.115.306130 26359510

[15] Nicolson PLR Nock SH Hinds J Garcia-Quintanilla L Smith CW Campos J. Low Dose Btk Inhibitors Selectively Block Platelet Activation by CLEC-2. Haematologica (2021) 106(1):208–19. doi: 10.3324/haematol.2019.218545 PMC7776357 31949019

[16] Nicolson PLR Hughes CE Watson S Nock SH Hardy AT Watson CN. Inhibition of Btk by Btk-Specific Concentrations of Ibrutinib and Acalabrutinib Delays But Does Not Block Platelet Aggregation to GPVI. Haematologica (2018) 103(12):2097–108. doi: 10.3324/haematol.2018.193391 PMC6269309 30026342

[17] Series J Garcia C Levade M Viaud J Sié P Ysebaert L. Differences and Similarities in Ibrutinib and Acalabrutinib Effects on Platelet Functions. Haematologica (2019) 104(11):2292–9 doi: 10.3324/haematol.2018.207183 PMC6821604 30819914

[18] Dmitrieva EA Nikitin EA Ignatova AA Vorobyev VI Poletaev AV Seregina EA. Platelet Function and Bleeding in Chronic Lymphocytic Leukemia and Mantle Cell Lymphoma Patients on Ibrutinib. J Thromb Haemost (2020) 18:2672–84. doi: 10.1111/jth.14943 32511880

[19] Koupenova M Clancy L Corkrey HA Freedman JE. Circulating Platelets as Mediators of Immunity, Inflammation, and Thrombosis. Circ Res (2018) 122:337–51. doi: 10.1161/circresaha.117.310795 PMC5777300 29348254

[20] Ali RA Wuescher LM Worth RG. Platelets: Essential Components of the Immune System. Curr Trends Immunol (2015) 16:65–78. 27818580 PMC5096834

[21] Gaertner F Ahmad Z Rosenberger G Fan S Nicolai L Busch B. Migrating Platelets Are Mechano-Scavengers That Collect and Bundle Bacteria. Cell (2017) 171:1368–82.e23. doi: 10.1016/j.cell.2017.11.001 29195076

[22] Palankar R Kohler TP Krauel K Wesche J Hammerschmidt S Greinacher A. Platelets Kill Bacteria by Bridging Innate and Adaptive Immunity *via* Platelet Factor 4 and Fcγriia. J Thromb Haemost (2018) 16:1187–97. doi: 10.1111/jth.13955 29350833

[23] Wolff M Handtke S Palankar R Wesche J Kohler TP Kohler C. Activated Platelets Kill Staphylococcus Aureus, But Not Streptococcus Pneumoniae—The Role of Fcγriia and Platelet Factor 4/Heparinantibodies. J Thromb Haemost (2020) 18:1459–68. doi: 10.1111/jth.14814 32237268

[24] Kraemer BF Campbell RA Schwertz H Cody MJ Franks Z Tolley ND. Novel Anti-Bacterial Activities of β-Defensin 1 in Human Platelets: Suppression of Pathogen Growth and Signaling of Neutrophil Extracellular Trap Formation. PloS Pathog (2011) 7:e1002355. doi: 10.1371/journal.ppat.1002355 22102811 PMC3213094

[25] Dearden C. Disease-Specific Complications of Chronic Lymphocytic Leukemia. Hematology (2008) 2008:450–6. doi: 10.1182/asheducation-2008.1.450 19074125

[26] Haseeb M Anwar MA Choi S. Molecular Interactions Between Innate and Adaptive Immune Cells in Chronic Lymphocytic Leukemia and Their Therapeutic Implications. Front Immunol (2018) 9:2720. doi: 10.3389/fimmu.2018.02720 30542344 PMC6277854

[27] Tillman BF Pauff JM Satyanarayana G Talbott M Warner JL. Systematic Review of Infectious Events With the Bruton Tyrosine Kinase Inhibitor Ibrutinib in the Treatment of Hematologic Malignancies. Eur J Haematol (2018) 100:325–34. doi: 10.1111/ejh.13020 29285806

[28] Varughese T Taur Y Cohen N Palomba ML Seo SK Hohl TM. Serious Infections in Patients Receiving Ibrutinib for Treatment of Lymphoid Cancer. Clin Infect Dis (2018) 67:687–92. doi: 10.1093/cid/ciy175 PMC6093991 29509845

[29] Arman M Krauel K Tilley DO Weber C Cox D Greinacher A. Amplification of Bacteria-Induced Platelet Activation Is Triggered by Fcγriia, Integrin αiibβ3, and Platelet Factor 4. Blood (2014) 123:3166–74. doi: 10.1182/blood-2013-11-540526 PMC4023422 24642751

[30] Arman M Krauel K. Human Platelet IgG Fc Receptor Fcγriia in Immunity and Thrombosis. J Thromb Haemost (2015) 13:893–908. doi: 10.1111/jth.12905 25900780

[31] Watson CN Kerrigan SW Cox D Henderson IR Watson SP Arman M. Human Platelet Activation by Escherichia Coli: Roles for Fcγriia and Integrin αiibβ3. Platelets (2016) 27:1–6. doi: 10.3109/09537104.2016.1148129 27025455 PMC5000871

[32] Moriarty RD Cox A McCall M Smith SGJ Cox D. Escherichia Coli Induces Platelet Aggregation in an Fcγriia-Dependent Manner. J Thromb Haemost (2016) 14:797–806. doi: 10.1111/jth.13226 26669970

[33] Hamzeh-Cognasse H Damien P Chabert A Pozzetto B Cognasse F Garraud O. Platelets and Infections – Complex Interactions With Bacteria. Front Immunol (2015) 6:82. doi: 10.3389/fimmu.2015.00082 25767472 PMC4341565

[34] Kerrigan SW Poole A. Focusing on the Role of Platelets in Immune Defence Against Invading Pathogens. Platelets (2015) 26:285–5. doi: 10.3109/09537104.2015.1038230 25910074

[35] Oda A Ikeda Y Ochs HD Druker BJ Ozaki K Handa M. Rapid Tyrosine Phosphorylation and Activation of Bruton’s Tyrosine/Tec Kinases in Platelets Induced by Collagen Binding or CD32 Cross-Linking. Blood (2000) 95:1663–70. 10688822

[36] Goldmann L Duan R Kragh T Wittmann G Weber C Lorenz R. Oral Bruton Tyrosine Kinase Inhibitors Block Activation of the Platelet Fc Receptor CD32a (Fcγriia): A New Option in HIT? Blood Adv (2019) 3:4021–33. doi: 10.1182/bloodadvances.2019000617 PMC6963242 31809536

[37] Krauel K Pötschke C Weber C Kessler W Fürll B Ittermann T. Platelet Factor 4 Binds to Bacteria, Inducing Antibodies Cross-Reacting With the Major Antigen in Heparin-Induced Thrombocytopenia. Blood (2011) 117:1370–8. doi: 10.1182/blood-2010-08-301424 20959601

[38] Allen RD Zacharski LR Widirstky ST Rosenstein R Zaitlin LM Burgess DR. Transformation and Motility of Human Platelets: Details of the Shape Change and Release Reaction Observed by Optical and Electron Microscopy. J Cell Biol (1979) 83:126–42. doi: 10.1083/jcb.83.1.126 PMC2110449 511936

[39] Rigg RA Aslan JE Healy LD Wallisch M Thierheimer MLD Loren CP. Oral Administration of Bruton’s Tyrosine Kinase Inhibitors Impairs GPVI-Mediated Platelet Function. Am J Physiol-Cell Ph (2016) 310:C373–80. doi: 10.1152/ajpcell.00325.2015 PMC4971826 26659727

[40] Dobie G Kuriri FA Omar MMA Alanazi F Gazwani AM Tang CPS. Ibrutinib, But Not Zanubrutinib, Induces Platelet Receptor Shedding of GPIb-IX-V Complex and Integrin αiibβ3 in Mice and Humans. Blood Adv (2019) 3:4298–311. doi: 10.1182/bloodadvances.2019000640 PMC6929381 31869418

[41] Bose P Gandhi VV Keating MJ. Pharmacokinetic and Pharmacodynamic Evaluation of Ibrutinib for the Treatment of Chronic Lymphocytic Leukemia: Rationale for Lower Doses. Expert Opin Drug Met (2016) 12:1381–92. doi: 10.1080/17425255.2016.1239717 PMC5391303 27686109

[42] Byrd JC Harrington B O’Brien S Jones JA Schuh A Devereux S. Acalabrutinib (ACP-196) in Relapsed Chronic Lymphocytic Leukemia. N Engl J Med (2016) 374:323–32. doi: 10.1056/nejmoa1509981 PMC4862586 26641137

[43] Podoll T Pearson PG Evarts J Ingallinera T Bibikova E Sun H. Bioavailability, Biotransformation, and Excretion of the Covalent BTK Inhibitor Acalabrutinib in Rats, Dogs, and Humans. Drug Metab Dispos (2018) 47(2):145–54. doi: 10.1124/dmd.118.084459 30442651

[44] Bye AP Unsworth AJ Desborough MJ Hildyard CAT Appleby N Bruce D. Severe Platelet Dysfunction in NHL Patients Receiving Ibrutinib Is Absent in Patients Receiving Acalabrutinib. Blood Adv (2017) 1:2610–23. doi: 10.1182/bloodadvances.2017011999 PMC5728643 29296914

[45] Burkhart JM Vaudel M Gambaryan S Radau S Walter U Martens L. The First Comprehensive and Quantitative Analysis of Human Platelet Protein Composition Allows the Comparative Analysis of Structural and Functional Pathways. Blood (2012) 120:e73–82. doi: 10.1182/blood-2012-04-416594 22869793

[46] Nore BF Mattsson PT Antonsson P Bäckesjö C-M Westlund A Lennartsson J. Identification of Phosphorylation Sites Within the SH3 Domains of Tec Family Tyrosine Kinases. Biochim Et Biophys Acta Bba - Proteins Proteom (2003) 1645:123–32. doi: 10.1016/s1570-9639(02)00524-1 12573241

[47] Conley ME Dobbs AK Farmer DM Kilic S Paris K Grigoriadou S. Primary B Cell Immunodeficiencies: Comparisons and Contrasts. Annu Rev Immunol (2009) 27:199–227. doi: 10.1146/annurev.immunol.021908.132649 19302039

[48] Ali RA Wuescher LM Dona KR Worth RG. Platelets Mediate Host Defense Against Staphylococcus Aureus Through Direct Bactericidal Activity and by Enhancing Macrophage Activities. J Immunol (2017) 198:344–51. doi: 10.4049/jimmunol.1601178 PMC5173399 27895175

[49] Ford I Douglas CWI Cox D Rees DGC Heath J Preston FE. The Role of Immunoglobulin G and Fibrinogen in Platelet Aggregation by Streptococcus Sanguis. Brit J Haematol (1997) 97:737–7446. doi: 10.1046/j.1365-2141.1997.1342950.x 9217171

[50] McNicol A Zhu R Pesun R Pampolina C Jackson E Bowden G. A Role for Immunoglobulin G in Donor-Specific Streptococcus Sanguis-Induced Platelet Aggregation. Thromb Haemostasis (2006) 95:288–93. doi: 10.1160/th05-07-0491 16493491

[51] Harrison P Mackie I Mumford A Briggs C Liesner R Winter M. Haematology BC for S in. Guidelines for the Laboratory Investigation of Heritable Disorders of Platelet Function. Brit J Haematol (2011) 155:30–44. doi: 10.1111/j.1365-2141.2011.08793.x 21790527

[52] Ishdorj G Streu E Lambert P Dhaliwal HS Mahmud SM Gibson SB. IgA Levels at Diagnosis Predict for Infections, Time to Treatment, and Survival in Chronic Lymphocytic Leukemia. Blood Adv (2019) 3:2188–98. doi: 10.1182/bloodadvances.2018026591 PMC6650736 31324639

[53] Warmerdam PA van de Winkel JG Vlug A Westerdaal NA Capel PJ. A Single Amino Acid in the Second Ig-Like Domain of the Human Fc Gamma Receptor II Is Critical for Human IgG2 Binding. J Immunol Baltim Md 1950 (1991) 147:1338–43. 1831223

[54] Tomiyama Y Kunicki TJ Zipf TF Ford SB Aster RH. Response of Human Platelets to Activating Monoclonal Antibodies: Importance of Fc Gamma RII (CD32) Phenotype and Level of Expression. Blood (1992) 80:2261–8. doi: 10.1182/blood.V80.9.2261.2261 1421396

[55] Rosenfeld SI Ryan DH Looney RJ Anderson CL Abraham GN Leddy JP. Human Fc Gamma Receptors: Stable Inter-Donor Variation in Quantitative Expression on Platelets Correlates With Functional Responses. J Immunol Baltim Md 1950 (1987) 138:2869–73. 2952726

[56] Qiao J Schoenwaelder SM Mason KD Tran H Davis AK Kaplan ZS. Low Adhesion Receptor Levels on Circulating Platelets in Patients With Lymphoproliferative Diseases Before Receiving Navitoclax (ABT-263). Blood (2013) 121:1479–81. doi: 10.1182/blood-2012-12-467415 23429990

[57] Luu S Gardiner EE Andrews RK. Bone Marrow Defects and Platelet Function: A Focus on MDS and CLL. Cancers (2018) 10:147. doi: 10.3390/cancers10050147 PMC5977120 29783667

[58] Au AE Josefsson EC. Regulation of Platelet Membrane Protein Shedding in Health and Disease. Platelets (2016) 28:1–12. doi: 10.1080/09537104.2016.1203401 27494300

[59] Krijgsveld J Zaat SAJ Meeldijk J van Veelen PA Fang G Poolman B. Thrombocidins, Microbicidal Proteins From Human Blood Platelets, Are C-Terminal Deletion Products of CXC Chemokines. J Biol Chem (2000) 275:20374–81. doi: 10.1074/jbc.275.27.20374 10877842

[60] Coutre SE Byrd JC Hillmen P Barrientos JC Barr PM Devereux S. Long-Term Safety of Single-Agent Ibrutinib in Patients With Chronic Lymphocytic Leukemia in 3 Pivotal Studies. Blood Adv (2019) 3:1799–807. doi: 10.1182/bloodadvances.2018028761 PMC6595265 31196847

[61] Sun C Tian X Lee YS Gunti S Lipsky A Herman SEM. Partial Reconstitution of Humoral Immunity and Fewer Infections in Patients With Chronic Lymphocytic Leukemia Treated With Ibrutinib. Blood (2015) 126:2213–9. doi: 10.1182/blood-2015-04-639203 PMC4635117 26337493

[62] Byrd JC Hillmen P O’Brien S Barrientos JC Reddy NM Coutre S. Long-Term Follow-Up of the RESONATE Phase 3 Trial of Ibrutinib vs Ofatumumab. Blood (2019) 133:2031–42. doi: 10.1182/blood-2018-08-870238 PMC6509542 30842083

[63] Maffei R Maccaferri M Arletti L Fiorcari S Benatti S Potenza L. Immunomodulatory Effect of Ibrutinib: Reducing the Barrier Against Fungal Infections. Blood Rev (2019) 40:100635. doi: 10.1016/j.blre.2019.100635 31699465

[64] Hopper M Gururaja T Kinoshita T Dean JP Hill RJ Mongan A. Relative Selectivity of Covalent Inhibitors Requires Assessment of Inactivation Kinetics and Cellular Occupancy: A Case Study of Ibrutinib and Acalabrutinib. J Pharmacol Exp Ther (2019) 372:331–8. doi: 10.1124/jpet.119.262063. jpet.119.262063. 31871305

[65] Advani RH Buggy JJ Sharman JP Smith SM Boyd TE Grant B. Bruton Tyrosine Kinase Inhibitor Ibrutinib (PCI-32765) Has Significant Activity in Patients With Relapsed/Refractory B-Cell Malignancies. J Clin Oncol (2012) 31:88–94. doi: 10.1200/jco.2012.42.7906 23045577 PMC5505166

[66] Atkinson BT Ellmeier W Watson SP. Tec Regulates Platelet Activation by GPVI in the Absence of Btk. Blood (2003) 102:3592–9. doi: 10.1182/blood-2003-04-1142 12842985

[67] Rawat A Jindal AK Suri D Vignesh P Gupta A Saikia B. Clinical and Genetic Profile of X-Linked Agammaglobulinemia: A Multicenter Experience From India. Front Immunol (2021) 11:612323. doi: 10.3389/fimmu.2020.612323 33584693 PMC7873890

[68] Duraisingham SS Manson A Grigoriadou S Buckland M Tong CYW Longhurst HJ. Immune Deficiency: Changing Spectrum of Pathogens. Clin Exp Immunol (2015) 181:267–74. doi: 10.1111/cei.12600 PMC4516442 25677249

[69] Quek LS Bolen J Watson SP. A Role for Bruton’s Tyrosine Kinase (Btk) in Platelet Activation by Collagen. Curr Biol (1998) 8:1137–S1. doi: 10.1016/s0960-9822(98)70471-3 9778529

[70] Kerrigan SW. The Expanding Field of Platelet–Bacterial Interconnections. Platelets (2015) 26:293–301. doi: 10.3109/09537104.2014.997690 25734214

