# Supplementary material for: Bruton’s Tyrosine Kinase Inhibitors Impair FcγRIIA-Driven Platelet Responses to Bacteria in Chronic Lymphocytic Leukemia
Source: Front Immunol. 2021 Nov 29;12:766272. doi: 10.3389/fimmu.2021.766272 (PMC8667317; doi:10.3389/fimmu.2021.766272)
Supplement: Supplementary file 1 [file DataSheet_1.docx]

Supplementary Material

# Supplementary Methods

**Reagents**

Anti-FcγRIIA monoclonal antibody (mAb) IV.3 was obtained from BioXCell (New Hampshire, USA). Rabbit anti-mouse IgG F(ab')2 fragments and formaldehyde were from Thermo Fisher Scientific (Loughborough, UK). Crosslinked collagen-related peptide (CRP-xl) was from CambCol Laboratories (Ely, UK). Thrombin Receptor Activator Peptide 6 amide (TRAP-6) was from Bachem AG (St. Helens, UK). Human fibrinogen (plasminogen, von Willebrand Factor and fibronectin depleted) was from Enzyme Research Laboratories (Swansea, UK). Dasatinib was from Cell Guidance Systems (Cambridge, UK). Ibrutinib, acalabrutinib and prostacyclin (PGI_2_) were from Cayman Chemical (Michigan, USA). Eptifibatide (Integrilin) was a gift from Prof Steve Watson (University of Birmingham). TRITC phalloidin and Invitrogen ProLong^TM^ Diamond Antifade Mountant were from ThermoFisher Scientific (Massachusetts, USA). Other chemicals were from Merck (Sigma-Aldrich; Darmstadt, Germany) unless otherwise indicated.

**Bacterial culture and preparation**

*S. aureus* Newman was cultured anaerobically in Brain Hearth Infusion medium, and *E. coli* RS218 was cultured aerobically in LB medium, both at 37˚C overnight. Bacteria were washed and resuspended in PBS to an optical density (OD, 600nm) of 1.6, corresponding to 1 x10^9^ CFU/mL. In platelet aggregation experiments, bacterial suspensions were diluted 10-fold. For scavenging assays, bacterial suspensions were prepared to an OD_600nm_ of 1.0.

**Platelet preparation**

For platelet-rich plasma (PRP) preparation, sodium citrated blood was centrifuged at 190xg for 15 minutes, and the upper phase transferred to a fresh tube. To obtain platelet-poor plasma (PPP), an aliquot of PRP was centrifuged at 1700xg for 5 minutes and the platelet pellet discarded.

For washed platelets (WP) preparation, first PRP was obtained as above from whole blood in ACD. Citric acid (6mM) and apyrase (2U/ml) were added to PRP before being centrifuged at 800xg for 12 minutes. The resultant pellet was resuspended in wash buffer (36 mM citric acid, 10 mM EDTA, 5 mM D-glucose, 5 mM KCl, 90 mM NaCl) supplemented with apyrase (2U/ml) and centrifuged again. Pellets were then resuspended to 2.5 x10^8^/ml (for light transmission aggregometry) or 10 x10^8^/ml (for Phospho-Tec ELISA) in modified Tyrode’s buffer (20 mM HEPES, 134 mM NaCl, 2 mM KCl, 0.34 mM Na_2_HPO_4_, 12 mM NaHCO_3_, 1 mM MgCl_2_, 5 mM glucose) and supplemented with 1 mM CaCl_2_.

**Platelet factor 4 secretion**

Light transmission aggregometry reactions were stopped 10 minutes (for CRP-xl, TRAP-6 and ADP) or 20 minutes (for IV.3-xl or bacteria) after the start of aggregation was observed by addition of prostacyclin (4 µM). Samples were then centrifuged at 1500xg for 10 minutes at room temperature. Plasma supernatants were used for platelet factor 4 (PF4) enzyme-linked immunosorbent assay (ELISA) (Human CXCL4/ PF4 ELISA; R&D Systems).

**Platelet scavenging of bacteria**

Glass coverslips (12 mm ø) were coated in 0.005% poly-L-lysine for 15 minutes and washed with PBS. *S. aureus* Newman suspensions (OD_600nm_ of 1.0) were added to coverslips for 1 hour at room temperature before being washed with PBS to remove unbound bacteria. The coverslips were then blocked with 0.5% heat-denatured fatty acid free BSA for 1 hour and washed with PBS. PRP samples were diluted with modified Tyrode’s buffer and supplemented with autologous plasma as required to give a final concentration of 2 x10^7^ platelets/ml with 15% plasma. 200 µl of platelet suspension were added to the bacteria-coated coverslips and incubated at 37˚C for 1 hour. Unbound platelets were removed by gently washing with PBS. Coverslips were fixed with 4% paraformaldehyde for 10 minutes, permeabilized with 0.3% Triton X-100 for 5 minutes, stained with Hoechst 33342 and TRITC phalloidin for 20 minutes and mounted onto glass slides using Invitrogen ProLong^TM^ Diamond Antifade Mountant. Coverslips were imaged at random using a Zeiss Axio Observer fluorescence microscope equipped with ApoTome structured illumination, a 40x oil immersion objective and an AxioCam 506 camera (Zeiss, UK). For selected platelets, Z-stacks were taken where the upper and lower limits were set so that the selected platelet and the bacterial layer, respectively, were fully out of focus. Slices were set at 0.2 µm distances, with each Z-stack averaging 80 slices. Images were processed and analyzed blinded (except for Z-stacks) using ImageJ (NIH, USA) and Zen Pro software (Zeiss UK).

The number of bacteria scavenged by individual platelets (e.g., bacteria cluster size) was calculated as follows. First, the background intensity per pixel of each image was calculated by circling 20 random areas devoid of bacteria and dividing the raw intensity by the area (number of pixels) and averaging. Next, the average intensity of a bacterium in an image was calculated by circling 50 random individual bacteria, subtracting the corresponding background (number of pixels × background intensity per pixel) and averaging. Finally, each cluster associated to a platelet was circled, the background was subtracted as for the previous step and the corrected intensity of the cluster was divided by the average intensity of a bacterium to give the number of bacteria in the cluster. Only clusters containing 3 or more bacteria were considered as resulting from scavenging.

**Platelet spreading on fibrinogen**

Glass coverslips were coated with 100 μg/ml human fibrinogen for 1 hour at room temperature, washed with PBS, blocked with 0.5% heat-denatured fatty acid free BSA for 1 hour, and washed with PBS. Platelets were prepared, allowed to spread, processed and imaged as described in the scavenging assay omitting the Hoechst 33342.

**Flow cytometry**

The following mAbs against human platelet surface receptors and relative isotype controls were used: RPE-conjugated anti-CD41 (integrin αIIb) (clone 5B12; DAKO) and mouse IgG1 control (DAK-GO1; DAKO); PE-conjugated anti-GPVI (clone HY101; BD Pharmingen) and mouse IgG1κ control (clone MOPC-21; BD Pharmingen); FITC-conjugated anti-CD41 (clone P2; Beckman Coulter) and anti-CD32 (FcγRIIA) (clone IV.3; StemCell Technologies) with anti-CD4 (clone OKT4, a mouse IgG2b against an irrelevant antigen for platelets; Biolegend) as control.

PRP was diluted at 1 x10^7^ platelets/ml with modified Tyrode’s buffer and incubated with mAbs for 20 minutes at room temperature. Non-stained samples were processed in addition. Platelets were fixed in 0.45% formaldehyde on ice and analyzed immediately after fixation. Events were acquired on a BD LSR Fortessa cell analyzer (BD Biosciences), following manufacturer’s guidance, with a valid CS&T procedure in each experimental day. Because samples were collected over a 6-week period (including healthy donors in each acquisition day), settings were highly standardized against target reference values for each parameter, using the 8-peak Rainbow Calibration Particles (standard average size of 3.2 µm, BD Biosciences) (Supplementary figure 4). Typically, 10000 events in the platelet gate were acquired using the FACS Diva Software. Data was later analyzed using the FlowJo software (BD).

**Protein phosphorylation studies**

Platelet lysates for Btk phosphorylation assays were collected as previously described (Arman et al. Blood. 2014;123(20):3166–3174) with minor modifications. Aggregation reactions in PRP were stopped by addition of an equal volume of ice-cold PBS at 3 minutes after the start of aggregation, or a parallel time point in samples in which aggregation was inhibited. Samples were transferred to a fresh tube and immediately pelleted by centrifugation at 8500xg for 50 seconds at 4°C, and pellets were lysed with 1x lysis buffer to give a final concentration equivalent to 5 x10^8^ platelets/ml. Protein detection in whole cell lysates was done by SDS-PAGE and immunoblotting for total Btk (anti-Btk D3H5 rabbit mAb; Cell Signaling Technology, Massachusetts, USA) and phospho-Y223 Btk (anti-Btk phospho Y223 EP420Y rabbit mAb; Abcam, Cambridge, UK). Membranes were imaged and quantified by near-Infrared Western Blot Detection using a Li-Cor IRDye® 680RD Goat anti-Rabbit IgG secondary antibody (LI-COR Biosciences, Cambridge, UK) and a Licor Odyssey CLx scanner (LI-COR Biosciences) and accompanying software. As loading control, membranes were re-probed for GAPDH (anti-GAPDH 6C5 mouse mAb, Santa Cruz Biotechnology,Texas, USA) followed by Li-Cor IRDye® 680RD Goat anti-mouse IgG secondary antibody.

**Normal adult hematological and serum immunoglobulin ranges**

Normal adult hematological reference ranges in whole blood are: Hb, 120-160 g/L for women and 135-175 g/L for men; WCC, 4-11 x10^9^/L; lymphocyte count, 0.8-3.4 x10^9^/L; platelet count, 150-400 x10^9^/L. Beta-2 microglobulin normal adult reference interval is 1.2-2.4 mg/L. Normal adult serum IgG concentration range is 6-16 g/L. Source: Hull University Teaching Hospitals NHS Trust (UK).

# Supplementary Data

# Supplementary Table 1. Concurrent medications taken by CLL patients enrolled in the study. Ibrutinib-untreated and treated CLL patients’ concurrent medications were compiled and classified by drug type based on the British National Formulary (Joint Formulary Committee. (2018). BNF 76^th^ Ed. Pharmaceutical Press). Frequency is presented as number of patients taking medications from that drug type (n) and percentage (%). Note that some patients were taking more than one medication per drug type.

| **Drug Type** | **Ibrutinib-untreated patients (n=34)** | | **Ibrutinib-treated patients (n=32)** | |
| --- | --- | --- | --- | --- |
| **Antidepressant** | 6 (18%) | Sertraline (2),  amitriptyline (2),  fluoxetine (1),  paroxetine (1) | 5 (16%) | Sertraline (1),  amitriptyline (4),  fluoxetine (1) |
| **Antidiarrheal Agent** | 1 (3%) | Loperamide (1) | 0 (0%) | - |
| **Hyperlipidemia**  **Statin** | 14 (41%) | Atorvastatin (7), simvastatin (7) | 8 (25%) | Atorvastatin (5), rosuvastatin (1),  simvastatin (1),  pravastatin (1) |
| **Hypertension** | 18 (53%) | Amlodipine (6),  ramipril (4),  perindopril (1),  bisoprolol (2), bendroflumethiazide (4), lisinopril (3),  enalapril (2),  lercanidipine (1), verapamil (1),  nifedipine (1),  angitil (1),  carvedilol (1),  atenolol (1),  losartan (3),  Indapamide (1) | 18 (56%) | Amlodipine (7),  ramipril (4),  perindopril (1),  bisoprolol (3),  lisinopril (2),  losartan (4),  entresto (1),  adipine (1),  carvedilol (1),  lercanidipine (1), indapamide (1) |
| **Erectile Dysfunction** | 2 (6%) | Sildenafil (2) | 1 (3%) | Sildenafil (1) |
| **Gastric and Duodenal Ulceration** | 9 (26%) | Omeprazole (4),  lansoprazole (5) | 14 (44%) | Omeprazole (3), lansoprazole (9),  ranitidine (3) |
| **Arthritis** | 0 (0%) | - | 1 (3%) | Etoricoxib (1) |
| **Osteoporosis** | 1 (3%) | Alendronate (1) | 1 (3%) | Alendronate (1) |
| **Anti-inflammatory** | 2 (6%) | Budesonide (1),  naproxen (1) | 0(0%) | - |
| **Vitamin and Iron Deficiency** | 5 (15%) | Calceos (2),  ferrous fumarate (1), accrete D3 (1),  thiamine (1) | 5 (16%) | Folic acid (2),  ferrous fumarate (2),  evacal D3 (1) |
| **Bladder and Urinary Disorders** | 6 (18%) | Tamsulosin (4),  doxazosin (2),  mirabegron (1) | 7 (22%) | Tamsulosin (2),  mirabegron (1),  solifenacin (1),  tolterodine (2),  finasteride (1),  alfuzosin (1) |
| **Hormone/Hormone Altering** | 3 (9%) | Levothyroxine (1), oestradiol (1),  finasteride (1) | 4 (13%) | Levothyroxine (3), dutasteride (1) |
| **Analgesics** | 6 (18%) | Co-codamol (4), paracetamol (2) | 14 (44%) | Co-codamol (2),  tramadol (2),  oxycodone (2),  codeine (3),  paracetamol (6),  ibuprofen (1) |
| **Antibiotic** | 0 (0%) | - | 19 (59%) | Amoxicillin (1),  co-trimoxazole (17), methenamine hippuarate (1) |
| **Antihistamine** | 1 (3%) | Fexofenadine (1) | 2 (6%) | Fexofenadine (1), hydroxyzine (1) |
| **Coronary Vasodilator** | 1 (3%) | Isosorbate mononitrate (1) | 0 (0%) | - |
| **Sedative** | 1 (3%) | Zopiclone (1) | 0 (0%) | - |
| **Chemotherapy** | 0 (0%) | - | 4 (13%) | Venetoclax (4) |
| **Hyperuricaemia drugs** | 1 (3%) | Allupurinol (1) | 2 (6%) | Allupurinol (2) |
| **Antiplatelet** | 6 (18%) | Aspirin (2),  clopidogrel (3),  warfarin (1) | 2 (6%) | Aspirin (2) |
| **Anti-Vertigo/Motion Sickness** | 1 (3%) | Hyoscine (1) | 1 (3%) | Betahistine (1) |
| **Anti-Diabetic** | 6 (18%) | Sitagliptin (1),  metformin (5),  gliclazide (1),  dapagliflozin (1), irbesartan (1) | 1 (3%) | Sitagliptin (1) |
| **Anti-Malarial**  (muscle cramp treatment) | 1 (3%) | Quinine (1) | 2 (6%) | Quinine (2) |
| **Antithrombotic** | 5 (15%) | Apixaban (5) | 0 (0%) | - |
| **Epilepsy** | 2 (6%) | Sodium valproate (1), clonazepam (1), gabapentin (1) | 0 (0%) | - |
| **Anti-Lipemic** | 1 (3%) | Bezafibrate (1) | 0 (0%) | - |
| **Diuretic** | 4 (12%) | Furosemide (2), bumetanide (1), spironolactone (1) | 1 (3%) | Spironolactone (1) |
| **Gout** | 0 (0%) | - | 1 (3%) | Allopurinol (1) |
| **Laxative** | 0 (0%) | - | 1 (3%) | Laxido (1) |
| **Mucolytic** | 0 (0%) | - | 1 (3%) | Carbocysteine (1) |
| **Muscle Relaxant** | 1 (3%) | Methocarbamol (1) | 0 (0%) | - |
| **Anti-asthmatic** | 1 (3%) | Montelukast (1) | 0 (0%) | - |

**Supplementary Table 2. Number of concurrent medications in CLL patients**. The number of medications excluding ibrutinib in each patient group is shown.

| **Number of Drugs** | **Ibrutinib-untreated patients (n=34)** | | **Ibrutinib-treated patients (n=32)** | |
| --- | --- | --- | --- | --- |
|  | **No. Patients** | **Percentage** | **No. Patients** | **Percentage** |
| **0** | 7 | 20.69 | 0 | 0 |
| **1** | 4 | 11.76 | 6 | 18.75 |
| **2** | 5 | 14.71 | 5 | 15.63 |
| **3** | 5 | 14.71 | 1 | 3.13 |
| **4** | 2 | 5.88 | 5 | 15.63 |
| **5** | 3 | 8.82 | 7 | 21.86 |
| **6** | 3 | 8.82 | 3 | 9.36 |
| **7** | 2 | 5.88 | 2 | 6.25 |
| **8** | 1 | 2.94 | 2 | 6.25 |
| **9** | 2 | 5.88 | 1 | 3.13 |

**
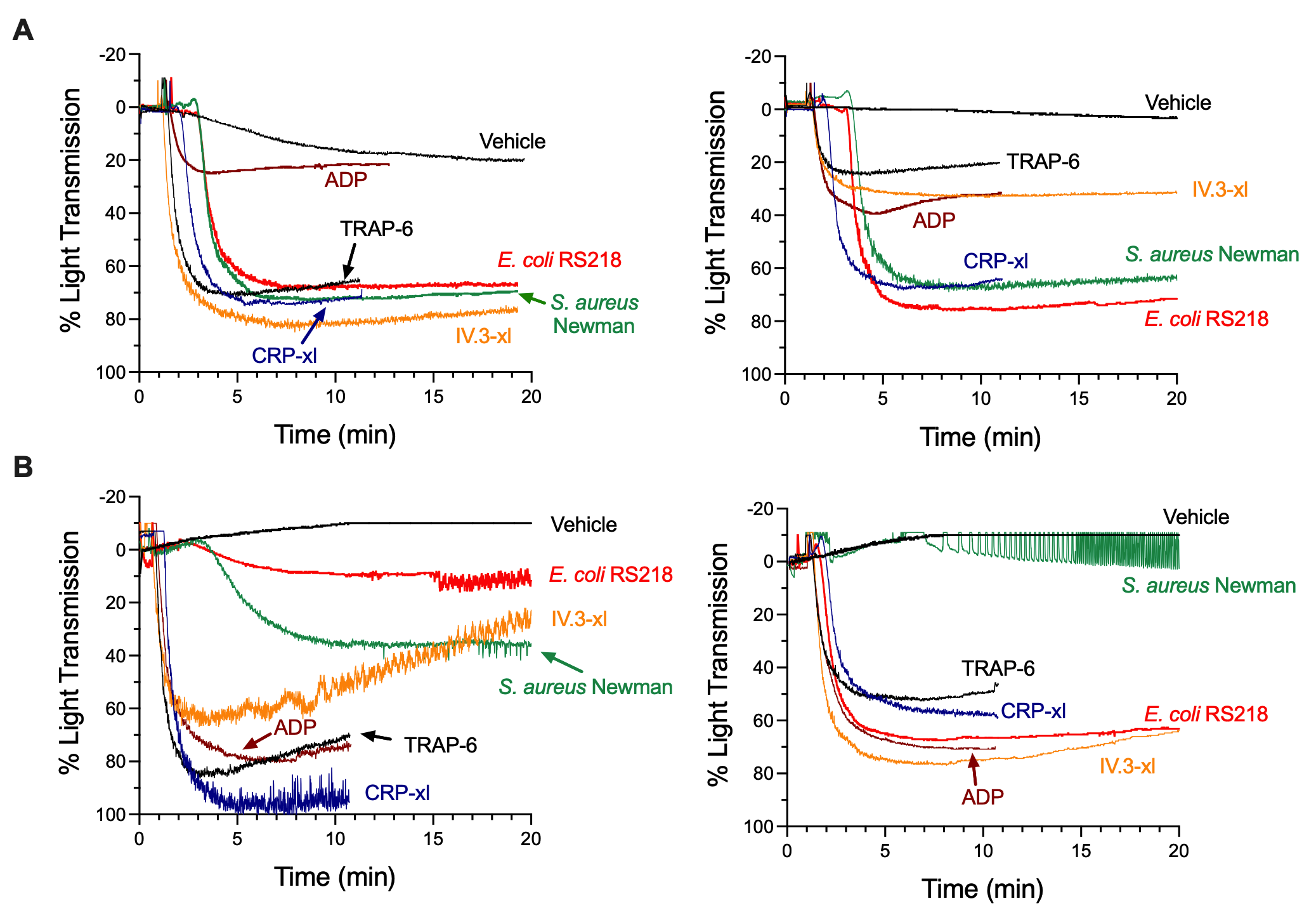
**

**Supplementary Figure 1. Platelet aggregation in CLL patients taking antiplatelet drugs.** PRP samples derived from ibrutinib-untreated CLL patients were stimulated with crosslinked IV.3 mAb (IV.3-xl; 4 μg/ml mAb IV.3 followed by 30 μg/ml F(ab')_2_ rabbit anti-mouse IgG), *S. aureus* Newman, *E. coli* RS218, 3 μg/ml CRP-xl, 3 μM TRAP-6, or 10 μM ADP. Aggregation was recorded by light transmission aggregometry. **(A)** Platelet aggregation results for two patients taking clopidogrel. **(B)** Platelet aggregation results for two patients taking aspirin. Only one ibrutinib-treated CLL patient was treated with concurrent aspirin therapy (data not shown); while this sample showed mild aggregation to TRAP-6 and ADP (49% and 56% respectively), responses to the other agonists were inhibited.


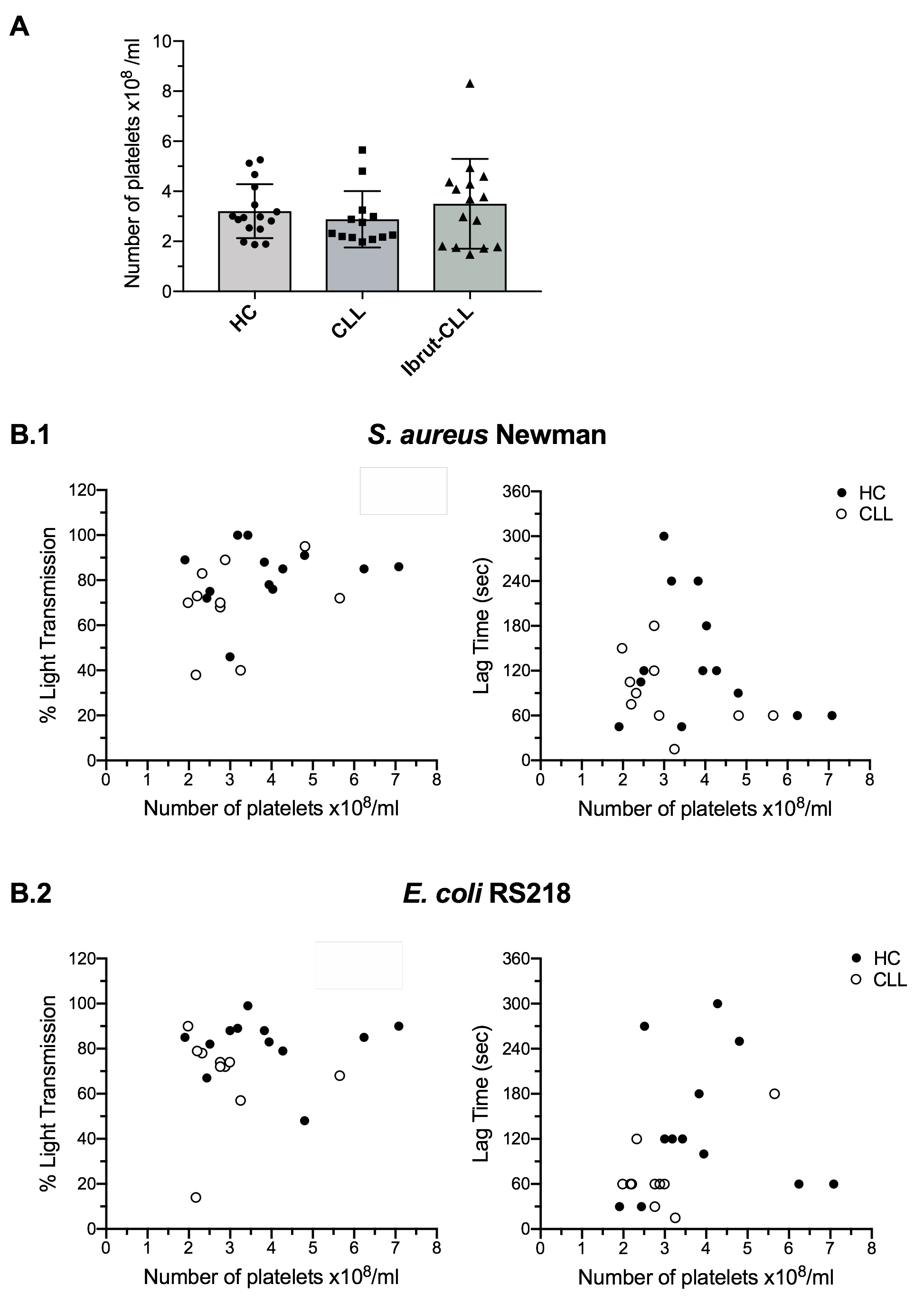


**Supplementary Figure 2. Platelet concentration in PRP samples from healthy donors and CLL patients. (A)** Platelet concentration of the PRP samples used for aggregation and PF4 release assays was measured using a Beckman Coulter Z1 particle counter. Data is shown as mean ± SD. **(B)** Scatter plots showing the relationship between platelet concentration and response to *S. aureus* Newman (B.1) and *E. coli* RS218 (B.2). Two-tailed Spearman correlation tests were performed with P ≤ 0.05 considered as significant. No correlation between number of platelets and maximum aggregation/lag time was detected in either healthy control or CLL samples except for CLL and lag time to *S. aureus* Newman induced aggregation (correlation coefficient = -0.68, p=0.034). HC, healthy controls; CLL, ibrutinib-untreated CLL patients, Ibrut-CLL, ibrutinib-treated CLL patients.


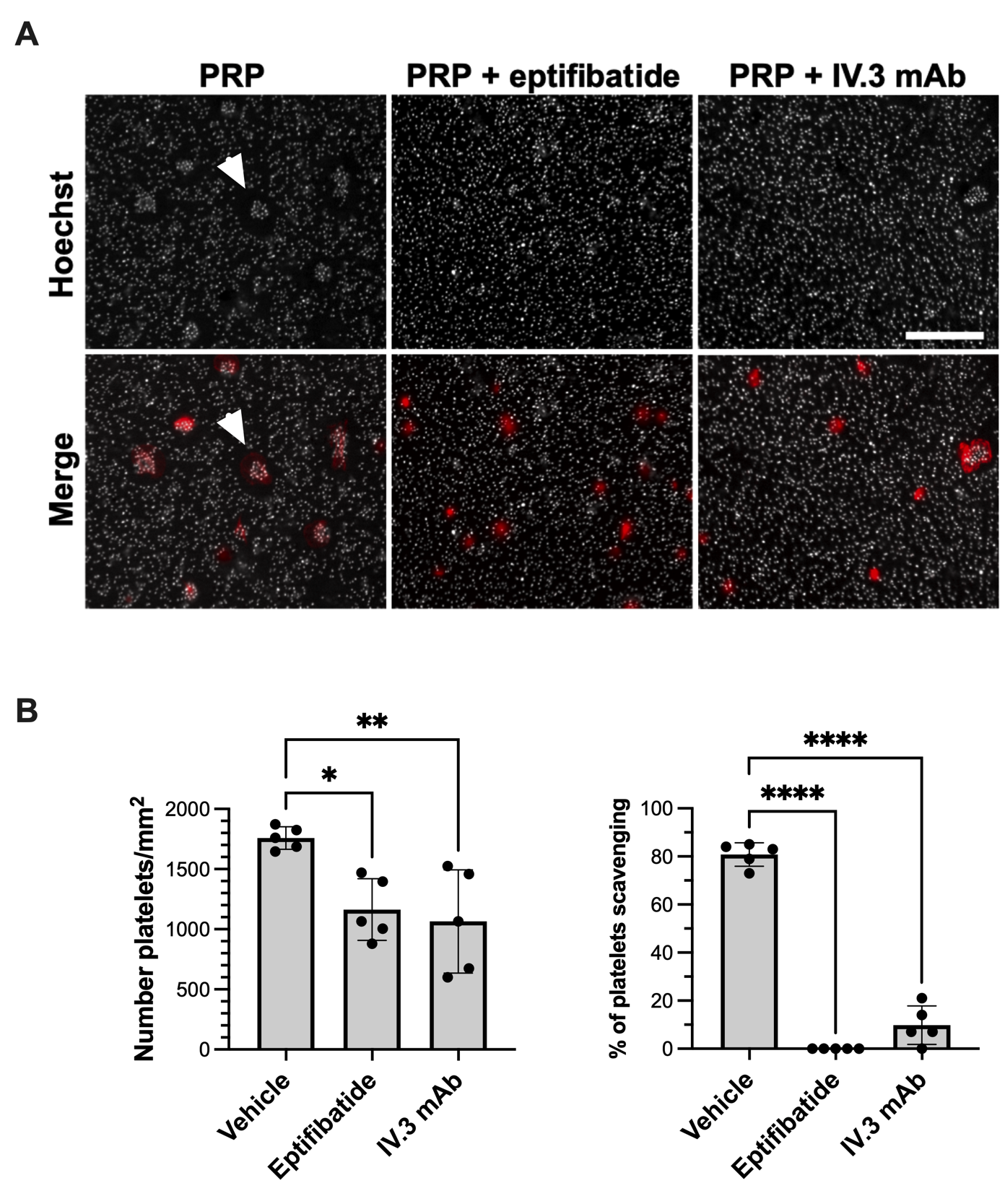


**Supplementary Figure 3. Platelet scavenging of *S. aureus* Newman is dependent on αIIbβ3 and FcγRIIA**. Healthy control PRP was adjusted to 1 x10^7^ platelets/ml and 5% final plasma with modified Tyrode’s buffer and autologous plasma. It was then incubated with eptifibatide (9 μM) or IV.3 mAb (15 μg/ml) for 2 min and 9 min, respectively. 200 μl of platelet suspension were then placed on coverslips containing immobilised *S. aureus* Newman and incubated at 37°C for 1 h. Cells were subsequently washed with PBS, fixed, permeabilized and incubated with a combination of 15 μg/ml Hoechst 33342 (grey color) and 1 μg/ml TRITC-phalloidin (red color on the merged image) for 30 min at room temperature. Coverslips were then mounted onto glass slides with ProLong Antifade Mountant and imaged at random using a Zeiss Axio Observer fluorescence microscope equipped with ApoTome structured illumination, a 40x oil immersion objective and an AxioCam 506 camera (Zeiss, UK). **(A)** Representative images of a total of five independent experiments are shown. Scale bar represents 20 μm. The arrowheads indicate an example of cluster of scavenged bacteria by a platelet. **(B)** The number of platelets per surface area (left panel) and the percentage of platelets scavenging bacteria (right panel) were determined as outlined in Material & Methods. Data is presented as mean ± SD of five independent experiments. Statistical significance was calculated using one-way ANOVA followed by Tukey’s multiple comparison correction (*p ≤0.05, **p ≤0.01, ****p ≤0.0001).


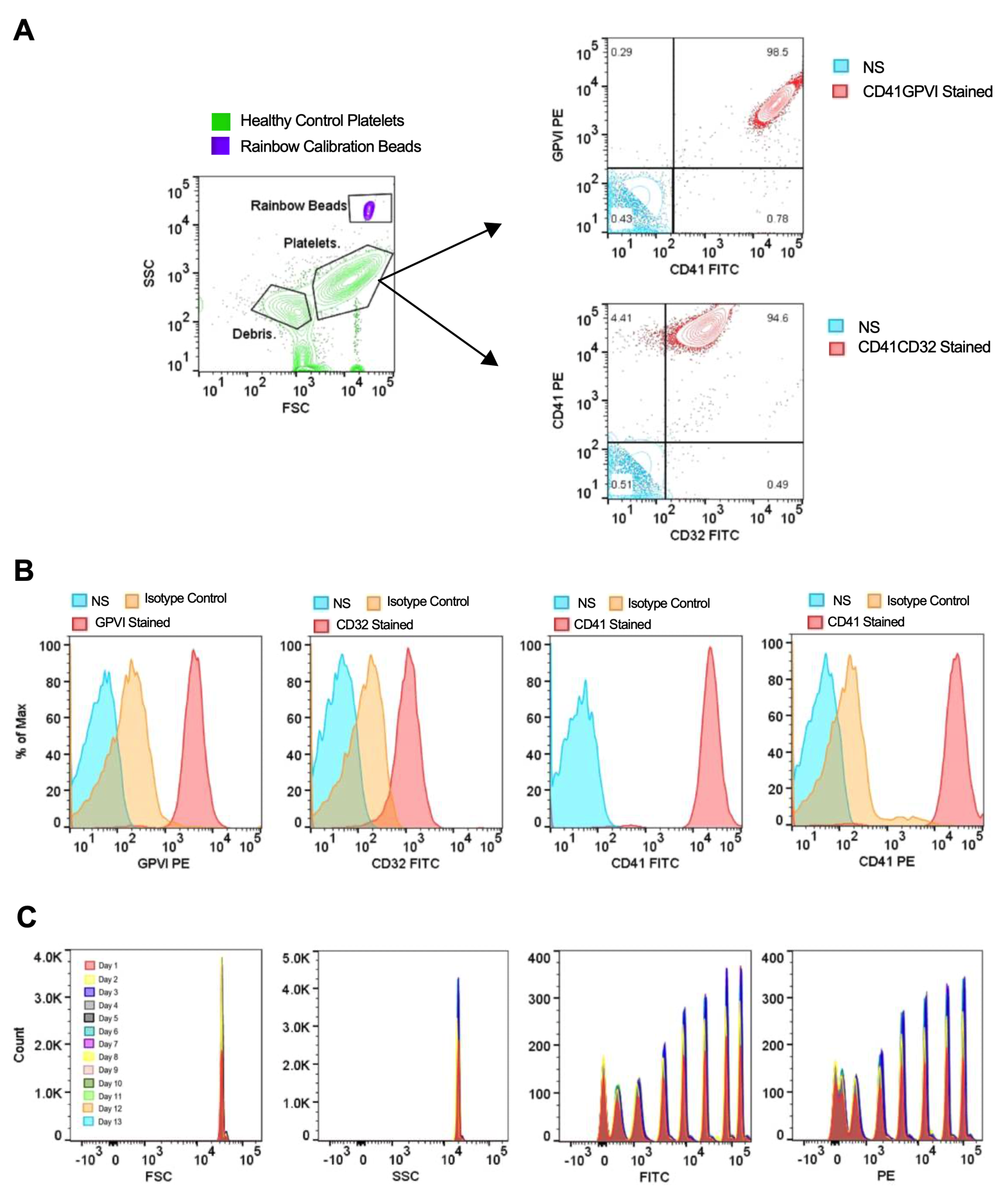


**Supplementary Figure 4**. **Experimental settings used for the detection of αIIbβ3, GPVI, and FcγRIIA platelet membrane expression by flow cytometry.**Human platelet surface expression of integrin αIIb (CD41), GPVI and FcγRIIa (CD32) was analyzed in PRP samples on a BD LSR Fortessa cell analyzer (BD Biosciences), as described in the Materials and Methods section. **(A)** Contour plots illustrate the gating strategy in a representative healthy donor sample. The left panel shows platelets (green, right) and debris (green, left) firstly identified based on physical parameters (size and granularity), compared to Rainbow Calibration beads (BD Biosciences) that have 3.2 µm standard average size (violet). On the right-hand side, overlaid contour plots show CD41-FITC/GPVI-PE (red, top) and CD41-PE/CD32-FITC (red, bottom) dual stains, compared to non-stained cells (blue), in gated platelets as indicated by black arrows. Two different antibody clones directed against platelet-specific CD41 marker were used to identify platelets. **(B)** Histogram plots show the overlay of non-stained (blue) samples with isotype control (orange) and platelet-antigen antibody (GPVI, CD32 and CD41; red) stains, in gated platelets from a representative donor. Refer to Figure 3B for the relative statistical validation. **(C)** Forward (FSC) and side (SSC) scatter, FITC and PE profiles were highly standardized across the study using the 8-peaks Rainbow Calibration beads to align parameters to target reference values, in any acquisition day over the 6-weeks period/experimental groups. A total of 13 flow cytometry acquisitions were performed over six weeks, overlaid in the histogram plots (color code in the left panel). Our settings allowed a highly accurate and reproducible detection of bead size (FSC), granularity (SSC) and any fluorescence (FITC and PE), as identified by perfectly aligned peaks during time. Such consistency in the rainbow profile removes the effects of daily instrument oscillations and allows biological differences to be detected/compared reliably, across time and experimental groups.


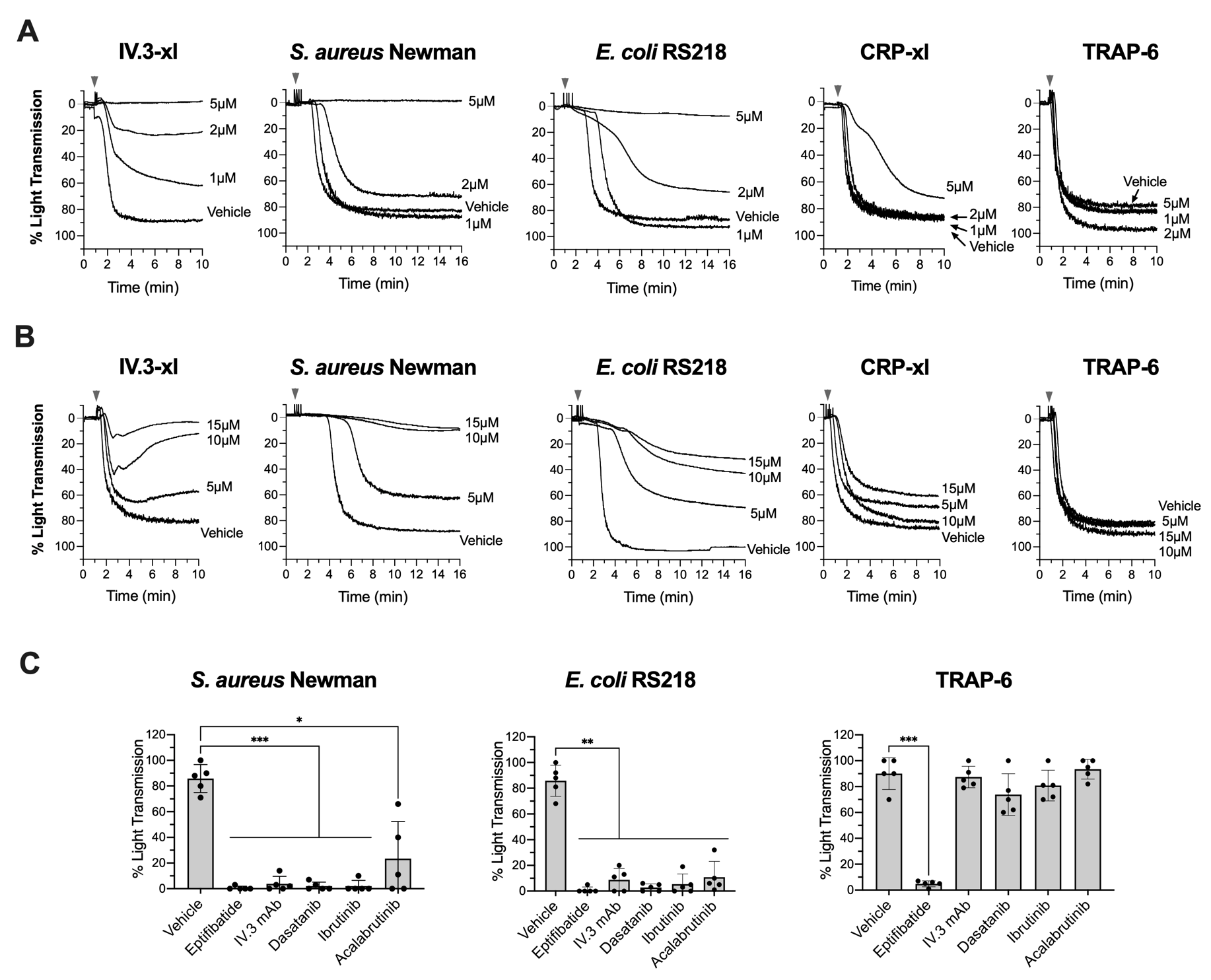


**Supplementary Figure 5**. **Ibrutinib and acalabrutinib *in vitro* treatments inhibit FcγRIIA-mediated platelet activation in the presence of plasma. (A)** Representative traces of light transmission aggregometry showing the effect of ibrutinib on platelet aggregation to assorted agonists in the presence of plasma (refer to Figure 4A for pooled data). Healthy control PRP samples were incubated with different doses of ibrutinib or vehicle for 5 minutes before agonist stimulation. Agonists included crosslinked IV.3 mAb (IV.3-xl; 4 μg/ml IV.3 mAb followed by 30 μg/ml F(ab')_2_ rabbit anti-mouse IgG), *S. aureus* Newman, *E. coli* RS218, 3 μg/ml CRP or 3 μM TRAP-6. Grey arrowheads indicate time of agonist injection. **(B)** Representative traces of light transmission aggregometry showing the effect of acalabrutinib on platelet aggregation to assorted agonists in the presence of plasma (refer to Figure 4B for pooled data). Healthy control PRP samples were incubated with different doses of acalabrutinib or vehicle for 5 minutes before agonist stimulation performed as above. **(C)** Healthy control PRP was incubated with either vehicle, 9 μM eptifibatide (αIIbβ3 inhibitor, 2 minutes), 20 μg/ml IV.3 mAb (FcγRIIA inhibitor, 10 minutes), 4 μM dasatinib (Src inhibitor, 2 minutes), or iBtks (5 minutes), 5 μM ibrutinib and 15 μM acalabrutinib before stimulation with stated agonists. Aggregation was measured by light transmission aggregometry. Reactions were run for 20 minutes from onset of aggregation for bacteria, and 10 minutes for TRAP-6. Data is shown as mean of maximum aggregation ± SD of five independent experiments. Statistical significance was calculated using one-way ANOVA followed by Tukey’s multiple comparison correction (*p ≤0.05, **p ≤0.01, ***p ≤0.001).


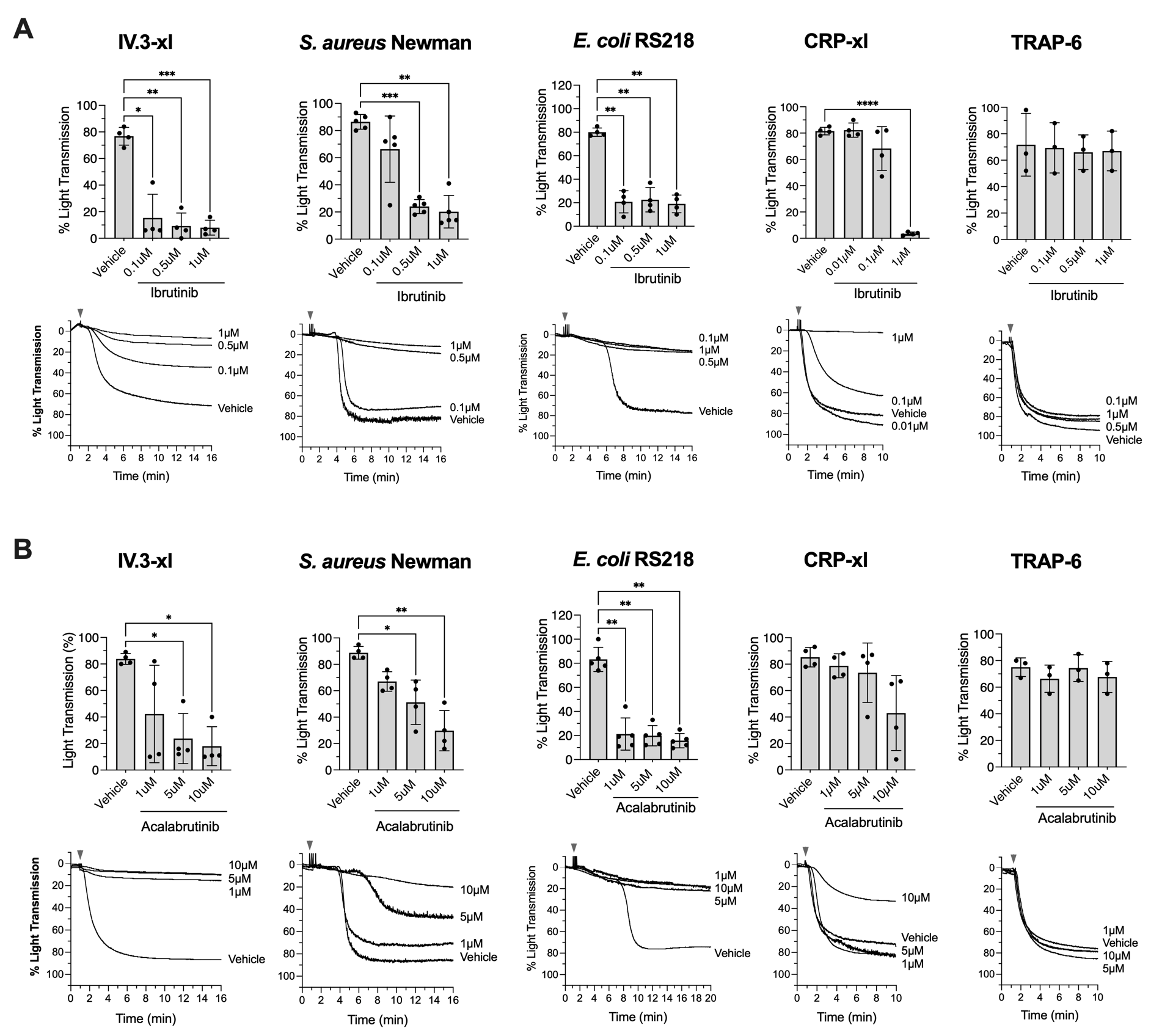


**Supplementary Figure 6**. **Ibrutinib and acalabrutinib *in vitro* treatments inhibit washed platelet activation by IV.3 mAb mediated FcγRIIA crosslinking and bacteria**. Healthy control washed platelets were adjusted to 2.5 x10^8^/ml, supplemented with 1 mg/ml fibrinogen and 0.2 mg/ml human IgG, and stimulated with either crosslinked IV.3 mAb (IV.3-xl; 4 μg/ml IV.3 followed by 30 μg/ml F(ab')_2_ rabbit anti-mouse IgG), *S. aureus* Newman, *E. coli* RS218, 3 μg/ml CRP or 3 μM TRAP-6 after incubation with either **(A)** ibrutinib, **(B)** acalabrutinib or vehicle at the stated concentrations for 5 minutes. Note that for CRP-xl, ibrutinib concentration 0.5 μM was not tested. Aggregation was measured by light transmission aggregometry. Reactions were run for 20 minutes from onset of aggregation for IV.3-xl and bacteria, and 10 minutes for the rest of agonists. Data on the top graphs is shown as mean of maximum aggregation ± SD of three to five independent experiments. Statistical significance was calculated using one-way ANOVA followed by Tukey’s multiple comparison correction (*p ≤0.05, **p ≤0.01). Representative traces of light transmission aggregation are shown below the pooled data graphs, with grey arrowheads indicating time of agonist injection.
